# Racial and Ethnic and Rural Variations in the Use of Hybrid Prenatal Care in the US

**DOI:** 10.1001/jamanetworkopen.2024.49243

**Published:** 2024-12-06

**Authors:** Peiyin Hung, Jiani Yu, Sayward E. Harrison, Jihong Liu, Adiba Promiti, Cassie Odahowski, Berry A. Campbell, Anirban Chatterjee, Nansi S. Boghossian, Bo Cai, Chen Liang, Jixuan Li, Xiaoming Li

**Affiliations:** 1 Rural and Minority Health Research Center, Arnold School of Public Health, University of South Carolina, Columbia; 2 Department of Health Services Policy & Management, Arnold School of Public Health, University of South Carolina, Columbia; 3 South Carolina SmartState Center for Healthcare Quality, University of South Carolina, Columbia; 4 Department of Population Health Sciences, Population Health Sciences, Weill Cornell Medical College, New York, New York; 5 Department of Psychology, College of Arts and Sciences, University of South Carolina, Columbia; 6 Department of Epidemiology and Biostatistics, Arnold School of Public Health, University of South Carolina, Columbia; 7 Division of Maternal-Fetal Medicine, University of South Carolina School of Medicine, Columbia; 8 Department of Health Promotion, Education, and Behavior, Arnold School of Public Health, University of South Carolina, Columbia

## Abstract

**Importance:**

Understanding whether there are racial and ethnic and residential disparities in prenatal telehealth uptake is necessary for ensuring equitable access and guiding implementation of future hybrid (ie, both telehealth and in-person) prenatal care.

**Objective:**

To assess temporal changes in individuals using hybrid prenatal care before and during the COVID-19 public health emergency (PHE) by race and ethnicity and residence location in the US.

**Design, Setting, and Participants:**

This retrospective cohort study analyzed electronic health record data of prenatal care visits from the National COVID Cohort Collaborative Data Enclave, comprising data from 75 health systems and freestanding institutes in all 50 US states. Data were analyzed on 349 682 nationwide pregnancies among 349 524 people who gave birth from June 1, 2018, through May 31, 2022. Multivariable generalized estimating equations were used to examine variations in receiving hybrid vs only in-person prenatal care. Data phenotyping and analysis occurred from June 13, 2023, to September 27, 2024.

**Exposures:**

Prenatal period overlap (never, partially, or fully overlapping) with the COVID-19 PHE, maternal race and ethnicity, and urban or rural residence.

**Main Outcomes and Measures:**

Hybrid vs in-person–only prenatal care.

**Results:**

Of 349 682 pregnancies (mean [SD] age, 29.4 [5.9] years), 59 837 (17.1%) were in Hispanic or Latino individuals, 14 803 (4.2%) in non-Hispanic Asian individuals, 65 571 (18.8%) in non-Hispanic Black individuals, 162 677 (46.5%) in non-Hispanic White individuals, and 46 794 (13.4%) in non-Hispanic individuals from other racial and ethnic groups. A total of 31 011 participants (8.9%) resided in rural communities. Hybrid prenatal care increased from nearly none before March 2020 to a peak of 8.1% telehealth visits in November 2020, decreasing slightly to 6.2% by March 2022. Among the fully overlapping group, urban residents had nearly 2-fold odds of hybrid prenatal care compared with rural people (adjusted odds ratio [AOR], 1.98; 95% CI, 1.84-2.12). Hispanic or Latino people (AOR, 1.48; 95% CI, 1.41-1.56), non-Hispanic Asian people (AOR, 1.47; 95% CI, 1.35-1.59), and non-Hispanic Black people (AOR, 1.18; 95% CI, 1.12-1.24) were more likely to receive hybrid prenatal care than non-Hispanic White people.

**Conclusions and Relevance:**

In this cohort study, hybrid prenatal care increased substantially during the COVID-19 PHE, but pregnant people living in rural areas had lower levels of hybrid care than urban people, and individuals who belonged to racial and ethnic minority groups were more likely to have hybrid care than White individuals. These findings suggest that strategies that improve equitable access to telehealth for people who live in rural areas and people in some minority racial and ethnic groups may be useful.

## Key Points

**Question:**

Did hybrid (ie, both telehealth and in-person) prenatal care uptake change and differ by maternal race and ethnicity and residence location after certain telehealth restrictions were waived during the COVID-19 pandemic?

**Findings:**

In this cohort study of 349 682 US pregnancies, hybrid prenatal care increased from nearly none in 2018 to a peak at 8.1% in November 2020, before decreasing to 6.2% by March 2022. Individuals who resided in urban areas and those who belonged to racial and ethnic minority groups were more likely to have hybrid care than were rural and non-Hispanic White individuals.

**Meaning:**

The findings of this study suggest that, although rates of hybrid prenatal care increased during the COVID-19 pandemic, particularly among Black and Hispanic individuals, overall rates remain low and the rural-urban gap persists.

## Teaser

This cohort study examines the use of combined telehealth and in-person prenatal care in pregnant individuals during the COVID-19 pandemic by race and ethnicity and by urban vs rural residence.

## Introduction

Prenatal care is crucial for the nearly 4 million individuals who give birth each year in the US.^1^ Barriers to accessing prenatal care can adversely impact maternal and neonatal health.^2,3,4^ Nationally, non-Hispanic Black (3.5%) and Hispanic (2.7%) pregnant people are more likely to receive no prenatal care compared with non-Hispanic White (1.4%) pregnant people.^2^ Some pregnant people, including those living in rural areas, need to travel longer distances to access prenatal care, leading to delayed prenatal care, lesser care, and/or poorer outcomes than those living in urban areas.^3,4,5,6^

Telehealth involving 2-way electronic communication services^7,8^ is endorsed by multiple federal agencies and professional associations, such as the US Department of Health and Human Services and the American College of Obstetricians and Gynecologists, for some aspects of prenatal care.^9,10^ It has been used to counsel patients, plan prenatal care teams, offer social and mental health support, read ultrasonography results, interpret laboratory results, manage pregnancy complications, and support remote patient monitoring for chronic disease management.^11,12^ Telehealth may also provide subspecialty maternity care that might not be locally accessible.^7,8,13^ In addition to potentially substituting for or complementing existing prenatal care services, the Coronavirus Response and Relief Supplemental Appropriations Act of 2021 emphasizes the use of telehealth to address rural-urban health care disparities.^14^ The Centers for Medicare and Medicaid Services (CMS) also included telehealth in the Transforming Maternal Health Model for providing whole-person care.^15^

During the COVID-19 public health emergency (PHE), the CMS allowed reimbursement of physicians for telehealth services and cross-state provision.^16^ Additionally, the Coronavirus Aid, Relief, and Economic Security (CARES) Act, signed March 2020, allocated $200 million for the COVID-19 Telehealth Program to support health care professionals in developing telehealth infrastructure.^16^

Despite the promise of telehealth for improving health care access,^17,18^ studies on prenatal telehealth uptake show mixed results regarding equitable use of telehealth among racial and ethnic groups.^19^ A national prepandemic study of commercially insured pregnant people found increased telehealth uptake for prenatal and postpartum care in Black and Hispanic pregnant people compared with non-Hispanic White people.^20^ However, during the COVID-19 PHE, prenatal telehealth use was lower in Black and/or Hispanic pregnant people in Tennessee and Colorado.^21,22^ Also, despite the wide telehealth adoptions and policy relaxations for originating sites and physician parity licensures during the COVID-19 PHE,^23^ telehealth uptake may be limited in rural areas due to insufficient broadband infrastructure, limited device access (eg, smartphones, tablets, and computers), and lower acceptability of leveraging telecommunication for health care.^24^ Understanding disparities in telehealth uptake is needed to develop targeted strategies for equity in care access, especially given previously documented regional variations in Medicare telemedicine use across hospital referral regions.^25^ Yet, national literature evaluating differences in prenatal telehealth use among diverse racial and ethnic and residential backgrounds is limited.

This study assessed temporal changes in telehealth- and in-person–delivered prenatal care before and during the COVID-19 PHE (June 2018 to May 2022) to compare telehealth uptake by race and ethnicity and residence location (urban and rural) in the US. We hypothesized that racial and ethnic and rural disparities in telehealth use for prenatal care existed before and were exacerbated by the COVID-19 PHE.

## Methods

### Data Sources and Study Design

This retrospective cohort study used multicenter electronic health record (EHR) data from the National COVID Cohort Collaborative (N3C) Data Enclave. The N3C cohort is constructed using phenotypic definitions approved by the N3C executive committee, encompassing all historical visit records and EHR data from the sampling timeframe, starting as early as January 2017, for patients who were COVID-19–positive and COVID-19–negative. Our analysis used these historical records, allowing us to include pregnancy data before and during the pandemic; therefore, their data would represent the general population who gave birth before the pandemic. The cohort included 349 682 childbirths (live births or stillbirths) to 349 524 unique birthing people who received prenatal care at 75 health systems and freestanding institutes across 1371 counties in 50 US states from June 1, 2018, through May 31, 2022. The N3C harmonizes EHR data of diverse standards using the Observational Medical Outcomes Partnership (OMOP) Common Data Model (CDM). Full details about the N3C data have been published.^26^ The University of South Carolina Institutional Review Board and the N3C Data Access Committee approved this study as an exempt study with a waiver of informed consent due to secondary data analyses. The study followed the Strengthening the Reporting of Observational Studies in Epidemiology (STROBE) reporting guideline for cohort studies.

Records of prenatal and intrapartum care from 75 health systems were identified using the OMOP CDM from the N3C (eTable 1 in Supplement 1). Pregnancy conception date was calculated by determining the estimated number of gestational weeks at the time of childbirth, converting these weeks into days (by multiplying by 7), and subtracting this date from the childbirth date. Each OMOP CDM concept and description^27^ was validated by 3 of us (P.H., A.C., and C.L.) and 1 obstetrician (B.A.C.). We extracted prenatal care data for each mother from prenatal care initiation to childbirth delivery date. These data were used to identify people who received prenatal care at the study health systems and, of those, how many had hybrid care (combining at least 1 telehealth visit and 1 in-person visit) vs only in-person care. The cleaned childbirth-level data were linked with patient enrollment data contributed by each health system for maternal age, self-reported race and ethnicity information, and residential information.

### Study Sample

**Figure 1.**
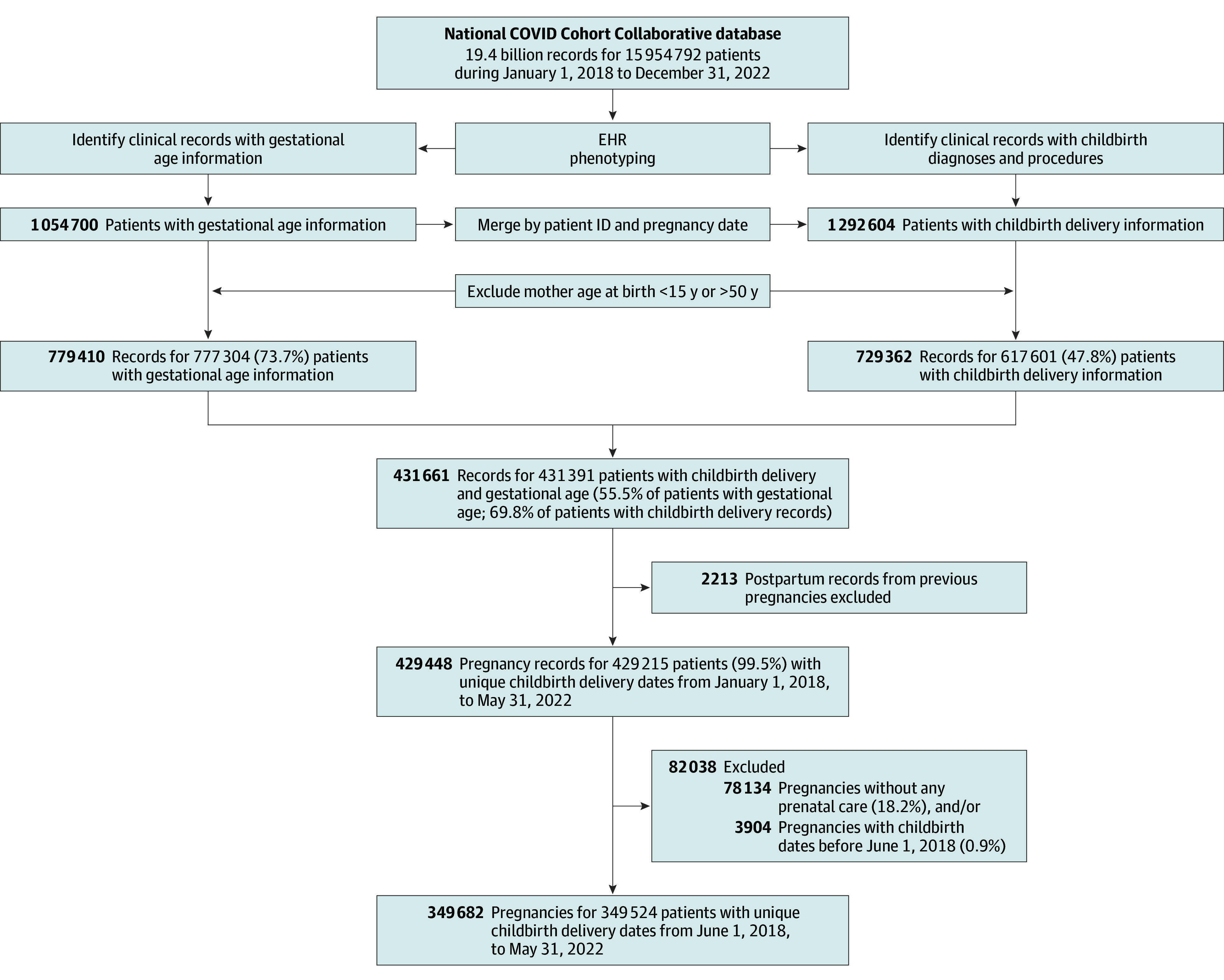
Sample Diagram Electronic health record (EHR) phenotyping involved identifying concept identification (ID) listed in eTable 1 in Supplement 1. Vocabulary classification and mapping of various ontologies to the Observational Medical Outcomes Partnership standard vocabulary is maintained by Observational Health Data Sciences and Informatics Network and publicly available on a web-based vocabulary repository.^27^

Figure 1 illustrates the sample selection of individuals receiving prenatal care from the N3C data.^27^ The study cohort included 349 682 pregnancies in 349 524 individuals aged 15 to 49 years who gave birth from June 1, 2018, through May 31, 2022. Individuals without residential county or zip code information in the EHR data (89 772 [25.7%]) were grouped into a separate stratum. When residential county was missing but the zip code was not, zip codes were used to assign individuals to their county of residence. For zip codes that crossed multiple counties, maternal residence was assigned to the county with the largest number of residents.

### Measures

The primary outcome—hybrid prenatal care—was defined as a pregnant person having had 1 or more telehealth encounter and 1 or more in-person encounter from estimated pregnancy conception to childbirth delivery date vs those who had only in-person prenatal care services. None of the pregnant persons in the study sample received exclusively telehealth prenatal care. Telehealth visits included the following modalities from the OMOP CDM: interactive video or audio communication, digital evaluation and management services, texting, asynchronous telecommunication platforms, and other concepts classified as telehealth and/or e-visits (eTable 2 in Supplement 1); however, telehealth visits for services other than prenatal care were not considered as prenatal telehealth uptake. Birthing individuals without prenatal care visits (1529 [0.4%]) were excluded from the analysis.

Exposure variables included COVID-19 PHE exposure during prenatal periods, maternal urban or rural residence, and race and ethnicity. We categorized pandemic exposure into 3 groups based on their prenatal period (eFigure 1 in Supplement 1): (1) no overlapping (births during June 2018-February 2020), (2) partially overlapping with the pandemic (conceived pregnancy before March 2020 and birth during or after March 2020), and (3) fully overlapping the pandemic (conceived pregnancy during or after March 2020). Urban and rural residence were defined at the county level using the 2023 United States Department of Agriculture’s Rural-Urban Continuum Codes.^28^

Self-reported maternal race and ethnicity was categorized as Hispanic or Latino (hereinafter, Hispanic), non-Hispanic Asian (hereinafter, Asian), non-Hispanic Black (hereinafter, Black), non-Hispanic White (hereinafter, White), and other groups (eg, multiple races, American Indian/Alaska Native, Native Hawaiian or Other Pacific Islander, or unknown).

To account for factors known to be associated with prenatal care use and technology acceptance,^29^ we included the following key covariates: maternal characteristics (age, prepregnancy body mass index classified as overweight or obese [≥25.0; calculated as weight in kilograms divided by height in meters squared], clinical conditions [gestational diabetes, preexisting diabetes, gestational hypertension, pregnancy-induced hypertension, multiple birth or singleton, and depressive or anxiety disorder during pregnancy]), health behaviors (smoking [eg, tobacco product, vaping] during pregnancy), gestational age at childbirth, and census region.

### Statistical Analysis

Descriptive statistics for maternal characteristics by pandemic group and by hybrid prenatal care were calculated and compared using Pearson χ^2^ or Fisher exact tests for categorical variables. Pregnancy-level number of prenatal care visits by pandemic exposure and maternal characteristics were compared using the Kruskal-Wallis tests. To assess the associations of maternal rural or urban residence location and race and ethnicity with hybrid prenatal care use, we applied the generalized estimating equation method with a logit link, controlling for the aforementioned covariates and clustering SEs within health systems. To further examine whether pandemic exposure (ie, never overlap, partial overlap, or full overlap with the pandemic) moderated racial and ethnic and rural or urban disparities in hybrid prenatal care use, we also included multiple 2-way interactions (ie, pandemic × race and ethnicity and pandemic × urban). Hybrid telehealth use differences by pandemic exposure were assessed through crude odds ratios (ORs) or adjusted ORs (AORs) and corresponding 95% CIs. All statistical analyses were performed within the N3C Enclave using R, version 4.0.2 (R Foundation for Statistical Computing), with 2-tailed statistical significance set at *P* < .05. Data phenotyping and analysis occurred from June 13, 2023, to September 27, 2024.

## Results

### Study Cohort

Of 349 682 pregnancies, 803 (4.2%) were in Asian individuals, 14 65 571 (18.8%) in Black individuals, 59 837 (17.1%) in Hispanic individuals, 162 677 (46.5%) in White individuals, and 46 794 (13.4%) in non-Hispanic individuals from other racial and ethnic groups (eTable 3 in Supplement 1). Among all races and ethnicities, most pregnant individuals were between the ages of 25 and 34 years (201 475 [57.6%]; mean [SD] age, 29.4 [5.9] years), had no prepregnancy overweight or obesity (242 419 [69.3%]), had a singleton birth (332 472 [95.1%]), and had a vaginal delivery (310 005 [88.7%]). A total of 31 011 participants (8.9%) resided in rural communities. Approximately 10% of pregnant patients smoked during pregnancy (242 419 [9.8%]) and/or had at least 1 SARS-CoV-2 infection during pregnancy (33 324 [9.5%]). Approximately 1 in 8 pregnant individuals had preexisting or gestational diabetes (12.0%), pregnancy-induced hypertension (15.3%), and depression and/or anxiety during pregnancy (15.4%).

### Prenatal Care Use by Prenatal Exposure to PHE

Overall, the study sample had a median of 14 (IQR, 6-22) visits (eTable 3 in Supplement 1), with 13 (IQR, 5-21) in-person prenatal care visits; among the telehealth users, the median was 2 (IQR, 1-4) telehealth visits. There was a slightly decreased number of prenatal care visits over time, from a median of 15 (IQR, 8-23) visits for those never exposed to the PHE to 13 (IQR, 6-22) visits for those partially and fully exposed to the PHE during pregnancy (*P* < .001) (Table 1). These decreases in total prenatal care visits coincided with an increased percentage of prenatal care visits via telehealth, from a mean (SD) of 0.4% (7.6%) in the never overlapping cohort to 2.2% (16.0%) among the fully overlapping cohort (eTable 4 in Supplement 1). Pregnancies never overlapping with the PHE had a mean of less than 1% prenatal care visits via telehealth (mean [SD], 0.4% [7.6%]), with 844 (0.9%) of pregnant people receiving any telehealth prenatal care. Among the partially overlapping cohort, a mean of 1.9% (12.7%) of prenatal care visits were via telehealth and 6254 (6.8%) pregnant people had telehealth prenatal care. Among the fully overlapping cohort, 2.2% (16.0%) of prenatal care visits were via telehealth and 14 213 (8.8%) people were fully exposed (Table 2; eFigure 2 in Supplement 1). Overall, prenatal care initiation was most common between 8 and 10 gestational weeks, while initial telehealth visits showed a peak slightly later, at 9 to 13 weeks (eFigure 3 in Supplement 1).

**Table 1.** Maternal Characteristics Before and During the COVID-19 PHE Among Birthing People With Prenatal Care

Characteristic	Pandemic exposure during pregnancy, overlapping, No (%)^a^^,^^b^		
	Never	Partially	Fully
No. (row %) of pregnancies	95 833 (27.4)	92 090 (26.3)	161 759 (46.3)
No. of prenatal care visits, median (IQR)	15 (8-23)	13 (6-22)	13 (6-22)
Residence			
Urban	64 025 (66.8)	57 312 (62.2)	10 7562 (66.5)
Rural	8129 (8.5)	7559 (8.2)	15 323 (9.5)
Missing	23 679 (24.7)	27 219 (29.6)	38 874 (24.0)
Maternal race and ethnicity			
Hispanic or Latino	13 877 (14.5)	16 482 (17.9)	29 478 (18.2)
Non-Hispanic group			
Asian	3518 (3.7)	4202 (4.6)	7083 (4.4)
Black	19 186 (20.0)	16 083 (17.5)	30 302 (18.7)
White	48 095 (50.2)	42 069 (45.7)	72 513 (44.8)
Other^c^	11 157 (11.6)	13 254 (14.4)	22 383 (13.8)
Mother’s age, y			
15-19	4879 (5.1)	4483 (4.9)	8574 (5.3)
20-24	16 812 (17.5)	14 676 (15.9)	27 152 (16.8)
25-29	26 373 (27.5)	24 407 (26.5)	42 985 (26.6)
30-34	29 543 (30.8)	28 854 (31.3)	49 313 (30.5)
35-39	15 154 (15.8)	15 992 (17.4)	26 832 (16.6)
40-49	3072 (3.2)	3678 (4.0)	6903 (4.3)
Prepregnancy BMI			
Underweight or healthy weight (<25.0)	68 770 (71.8)	65 073 (70.7)	108 576 (67.1)
Overweight or obesity (≥25.0)	27 063 (28.2)	27 017 (29.3)	53 183 (32.9)
Smoking during pregnancy	9939 (10.4)	9148 (9.9)	15 172 (9.4)
Preexisting or gestational diabetes	10 491 (10.9)	11 217 (12.2)	20 265 (12.5)
Preexisting and/or pregnancy-induced hypertension	14 032 (14.6)	12 878 (14.0)	26 457 (16.4)
Depression and/or anxiety during pregnancy	15 449 (16.1)	13 352 (14.5)	25 130 (15.5)
Plurality			
Singleton	91 275 (95.2)	87 767 (95.3)	153 430 (94.9)
Multiple	4558 (4.8)	4323 (4.7)	8329 (5.1)
Gestational age at childbirth, wk			
Very preterm (≤28)	1410 (1.5)	678 (0.7)	2546 (1.6)
Preterm (29-36)	9247 (9.6)	7789 (8.5)	18 007 (11.1)
Full term (≥37)	85 176 (88.9)	83 623 (90.8)	141 206 (87.3)
Prenatal SARS-CoV-2 Infection	0	5500 (6.0)	27 824 (17.2)
Mode of delivery			
Any cesarean delivery	25 628 (26.7)	25 287 (27.5)	46 006 (28.4)
Vaginal delivery only	70 205 (73.3)	66 803 (72.5)	115 753 (71.6)
Region			
Northeast	13 025 (13.6)	14 453 (15.7)	21 411 (13.2)
Midwest	28 632 (29.9)	22 924 (24.9)	41 945 (25.9)
South	31 464 (32.8)	29 128 (31.6)	59 862 (37.0)
West	4335 (4.5)	3328 (3.6)	8166 (5.0)
Unknown	18 377 (19.2)	22 257 (24.2)	30 375 (18.8)

Abbreviations: BMI, body mass index (calculated as weight in kilograms divided by height in meters squared); PHE, public health emergency.

^a^ Prenatal periods for pregnant people who gave birth before March 1, 2020, were considered never overlapping to the COVID-19 pandemic; those for individuals whose conception occurred before March 1, 2020, and gave birth on March 1, 2020, and onward were considered partially overlapping, and those whose conception occurred on or after March 1, 2020, were considered fully overlapping with the COVID-19 pandemic.

^b^ *P* values were calculated from Pearson χ^2^ tests for categorical variables and Kruskal-Wallis test for median differences. All findings were significant at *P* < .001.

^c^ The Other group includes individuals identifying as American Indian or Alaska Native, multiracial, Native Hawaiian or Other Pacific Islander, or those with unknown or unreported race and ethnicity.

**Table 2.** Telehealth Uptake for Prenatal Care Among Pregnant People Giving Birth From June 2018 to May 2022

Variable	Overlapping pregnancies with any prenatal telehealth care, No. (%)^a^^,^^b^		
	Never (n = 95 833 [27.4%])	Partial (n = 92 090 [26.3%])	Full (n = 161 759 [46.3%])
All pregnancies	844 (0.9)	6254 (6.8)	14 213 (8.8)
Urban/rural residence			
Urban	834 (1.3)	5220 (9.1)	11 991 (11.1)
Rural	<20 (0.1)	430 (5.7)	888 (5.8)
Missing	<20 (0.0)	604 (2.2)	1334 (3.4)
Race and ethnicity			
Hispanic or Latino	119 (0.9)	1333 (8.1)	2943 (10.0)
Non-Hispanic group			
Asian	27 (0.8)	340 (8.1)	814 (11.5)
Black	315 (1.6)	1284 (8.0)	3126 (10.3)
White	336 (0.7)	2656 (6.3)	5933 (8.2)
Other^c^	47 (0.4)	641 (4.8)	1397 (6.2)
Maternal age, y			
15-19	57 (1.2)	214 (4.8)	530 (6.2)
20-24	164 (1.0)	874 (6.0)	1862 (6.9)
25-29	237 (0.9)	1624 (6.7)	3484 (8.1)
30-34	241 (0.8)	2094 (7.3)	4713 (9.6)
35-39	118 (0.8)	1153 (7.2)	2827 (10.5)
40-49	27 (0.9)	295 (8.0)	797 (11.5)

^a^ Prenatal periods for pregnant people who gave birth before March 1, 2020, were considered never overlapping to the COVID-19 pandemic; those for individuals whose conception occurred before March 1, 2020, and gave birth on or after March 1, 2020, were considered partially overlapping, and those whose conception occurred on or after March 1, 2020, were considered fully overlapping with the COVID-19 pandemic.

^b^ *P* values were calculated to compare telehealth uptake status (hybrid vs in-person only) by pandemic exposures within each group of residence rurality, maternal race and ethnicity, and age using Pearson χ^2^ or Fisher exact tests. All findings were significant at *P* < .001.

^c^ The Other group includes individuals identifying as American Indian or Alaska Native, multiracial, Native Hawaiian or Other Pacific Islander, or those with unknown or unreported race and ethnicity.

### Variations in Hybrid Prenatal Care by Maternal Characteristics

**Figure 2.**
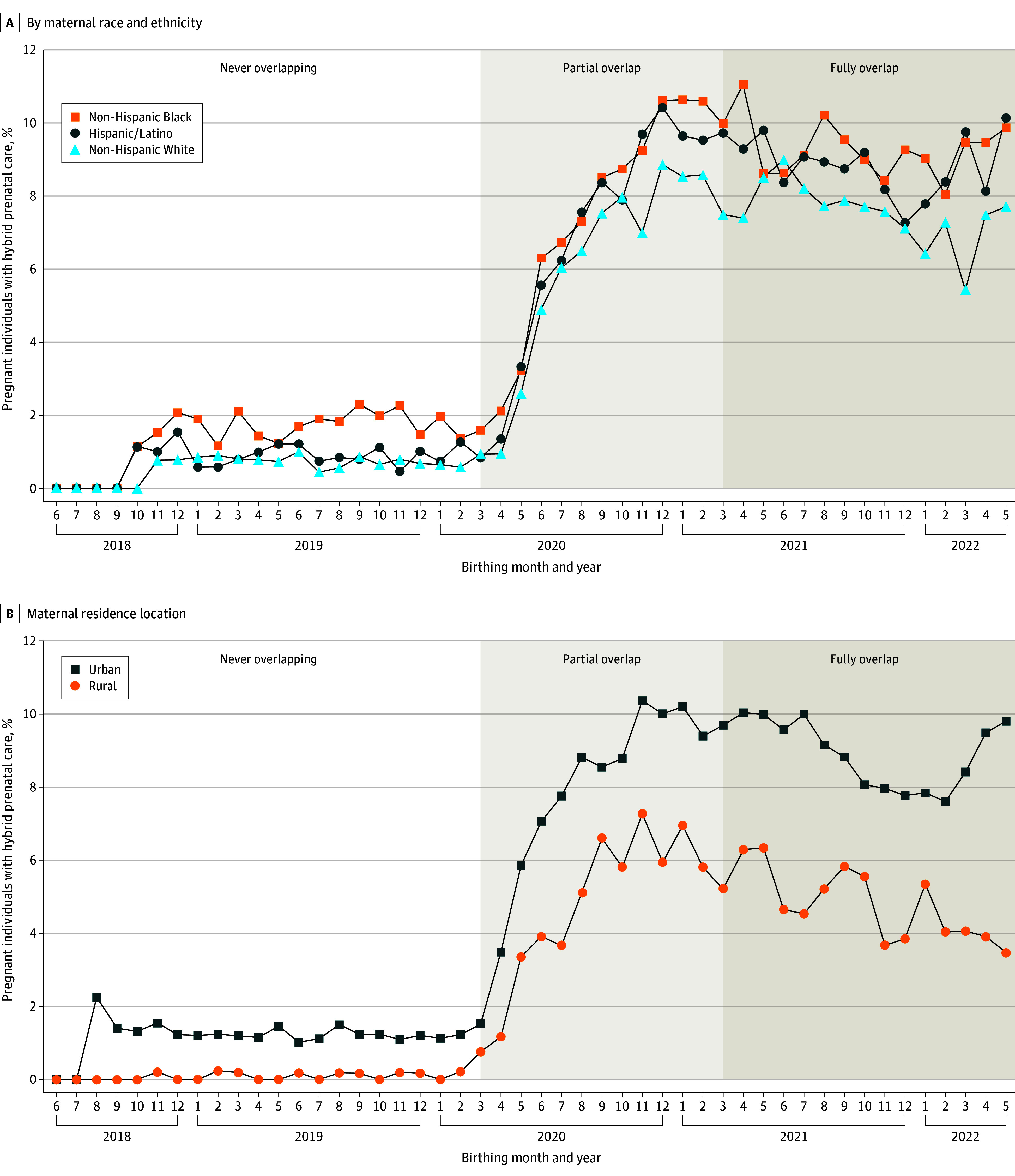
Trends in Percentage of Pregnant People Receiving Combined Telehealth and In-Person Prenatal Care, 2018-2022 Hybrid prenatal care was defined as at least 1 telehealth visit and 1 in-person visit for prenatal care. Residence location was categorized into urban or rural counties using the 2023 Rural-Urban Continuum Codes. The partial overlapping and fully overlapping periods are approximate as they were categorized based on a pregnancy-level prenatal exposure to the COVID-19 public health emergency (PHE). Prenatal periods for pregnant people who gave birth prior to March 1, 2020, were considered never overlapping to the COVID-19 PHE; those for individuals whose conception was before March 1, 2020, and gave birth on or after March 1, 2020, were considered partially overlapping, and those whose conception was on or after March 1, 2020, were considered fully overlapping to the COVID-19 PHE.

Over time, across all races and ethnicities and urban and rural residences, we observed a steady increase in hybrid prenatal care from March to July 2020, which peaked at 8.1% in November and eventually plateaued by December 2020, before experiencing a minor dip in December 2021 to February 2022 **(**Figure 2) and decreasing to 6.2% by March 2022. Nevertheless, the percentage of individuals with any telehealth use in both partially and fully exposed cohorts remained consistently higher (6%-11%) compared with the never-overlap cohort (<2%). Among those fully exposed to the PHE, Asian pregnant people had the highest rate of hybrid prenatal care use (11.5%; AOR, 1.47; 95% CI, 1.35-1.59), followed by Black people (10.3%; AOR, 1.18; 95% CI, 1.12-1.24), and Hispanic people (10.0%; AOR, 1.48; 95% CI, 1.41-1.56), while White people had lower rates of telehealth use (8.3%). Hybrid use was higher for urban residents but increased for both urban (from 0.9% in unexposed to 9.1% in partially exposed and 11.1% in fully exposed cohorts) and rural (from 0.1% in unexposed to 5.7% in partially exposed and 5.8% in fully exposed cohorts) pregnant people (Table 2). Among the fully overlapping group, urban residents had nearly 2-fold odds of hybrid prenatal care compared with rural people (adjusted odds ratio [AOR], 1.98; 95% CI, 1.84-2.12).

After controlling for other sociodemographic, behavioral, and clinical factors, the odds of hybrid prenatal care in pregnant individuals were increased in the partially exposed (AOR, 9.1; 95% CI, 8.4-9.7) (Table 3) and fully exposed (AOR, 11.6; 95% CI, 10.8-12.5) cohorts compared with those in the unexposed cohort. Urban compared with rural residence and Black race compared with White race differences in hybrid care use narrowed during the pandemic, compared with the differences seen in the never-overlapping cohort (eTable 5 in Supplement 1). Specifically, among the fully overlapping cohort, compared with White individuals, the odds of hybrid care were significantly higher in Asian (AOR, 1.5; 95% CI, 1.4-1.6), Black (AOR, 1.2; 95% CI, 1.1-1.2), and Hispanic (AOR, 1.5; 95% CI, 1.4-1.6) individuals. The odds of hybrid care in the other racial and ethnic group were significantly lower (AOR, 0.9; 95% CI, 0.8-1.0) than in the White group. Urban residents (AOR, 2.0; 95% CI, 1.8-2.1) compared with rural residents had higher odds of hybrid care (eTable 6 in Supplement 1). In addition, pregnant people with overweight or obesity (AOR, 1.4; 95% CI, 1.4-1.5) had preexisting or gestational diabetes (AOR, 1.4; 95% CI, 1.3-1.4), were diagnosed with anxiety and/or depression during pregnancy (AOR, 1.4; 95% CI, 1.3-1.4), smoked or vaped during pregnancy (AOR, 1.3; 95% CI, 1.3-1.4), or tested positive for SARS-CoV-2 (AOR, 1.1; 95% CI, 1.0-1.1) had significantly higher odds of hybrid care use compared with their peers without each condition.

**Table 3.** Maternal Characteristics Associated With Combined Telehealth and In-Person Prenatal Care (Hybrid Care), 2018-2022

Characteristic	Hybrid prenatal care, OR (95% CI)		*P* value^a^
	Crude	Adjusted	
COVID-19 pandemic exposure during pregnancy			
Never overlapping	1 [Reference]	1 [Reference]	
Partially overlapping	8.2 (7.6-8.8)	9.1 (8.4-9.7)	<.001
Fully overlapping	10.8 (10.1-11.6)	11.6 (10.8-12.5)	<.001
Urban/rural residence			
Urban	1.9 (1.8-2.0)	1.9 (1.8-2.0)	<.001
Rural	1 [Reference]	1 [Reference]	
Missing	0.5 (0.5-0.5)	0.2 (0.2-0.2)	<.001
Maternal race and ethnicity			
Hispanic or Latino	1.4 (1.3-1.4)	1.5 (1.4-1.5)	<.001
Non-Hispanic			
Asian	1.5 (1.4-1.6)	1.4 (1.3-1.5)	<.001
Black	1.3 (1.3-1.4)	1.2 (1.2-1.3)	<.001
White	1 [Reference]	1 [Reference]	
Other^b^	0.8 (0.8-0.8)	0.9 (0.8-0.9)	<.001
Mother’s age, y			
15-19	0.8 (0.7-0.8)	0.8 (0.8-0.9)	<.001
20-24	0.9 (0.8-0.9)	0.9 (0.8-0.9)	<.001
25-29	1 [Reference]	1 [Reference]	
30-34	1.2 (1.1-1.2)	1.1 (1.1-1.2)	<.001
35-39	1.3 (1.2-1.3)	1.2 (1.2-1.3)	<.001
40-49	1.5 (1.4-1.6)	1.2 (1.2-1.3)	<.001
Overweight or obesity^c^	1.8 (1.7-1.8)	1.5 (1.5-1.6)	<.001
Preexisting/gestational diabetes	1.8 (1.7-1.8)	1.4 (1.3-1.4)	<.001
Multiple birth vs singleton	0.6 (0.5-0.6)	0.6 (0.6-0.7)	<.001
Preexisting and/or pregnancy-induced hypertension	1.3 (1.3-1.4)	1.1 (1.0-1.1)	.008
Depression and anxiety during pregnancy	1.4 (1.3-1.4)	1.3 (1.2-1.3)	<.001
Smoking (eg, tobacco, vaping) during pregnancy	1.4 (1.3-1.4)	1.4 (1.3-1.5)	<.001
Gestational age at childbirth			
Very preterm (≤28 wk)	1 [Reference]	1 [Reference]	
Preterm (29-36 wk)	1.2 (1.1-1.4)	1.0 (0.9-1.2)	.93
Full term (≥37 wk)	1.3 (1.1-1.5)	1.1 (0.1-1.3)	.09
Region			
Northeast	1.6 (1.5-1.6)	1.5 (1.4-1.5)	<.001
Midwest	0.9 (0.9-1.0)	1.2 (1.1-1.2)	<.001
South	1 [Reference]	1 [Reference]	
West	0.6 (0.5-0.6)	0.6 (0.5-0.6)	<.001
Unknown	0.4 (0.4-0.4)	NA^d^	<.001

Abbreviations: NA, not applicable; OR, odds ratio.

^a^ *P* values and adjusted ORs were calculated from the generalized estimating equation with a binomial distribution and logit-link function at pregnancy level of receiving hybrid prenatal care (combined telehealth and in-person prenatal visits) vs all prenatal care visits via in-person care. 95% CIs were calculated by clustering SEs within health systems.

^b^ The other group includes individuals identifying as American Indian or Alaska Native, multiracial, Native Hawaiian or Other Pacific Islander, or those with unknown or unreported race and ethnicity.

^c^ Body mass index of 25.0 or higher (calculated as weight in kilograms divided by height in meters squared).

^d^ No within-health-system variations.

## Discussion

In this national cohort of pregnant people, we found that the odds of hybrid prenatal care increased 10-fold at the peak of the early COVID-19 PHE. Hybrid prenatal care remained consistently higher as of May 2022 compared with the prepandemic period; however, only about 1 in 10 pregnant individuals were receiving prenatal telehealth services—even at peak use. Additionally, we found that none of the pregnant individuals in this study received exclusively telehealth services for prenatal care, suggesting that telehealth functions primarily as a complement to in-person services rather than a substitute. While our study found relatively higher telehealth uptake among racial and ethnic minority groups who historically experienced inadequate prenatal care, such as Black and Hispanic individuals compared with White individuals, rural residents were less likely to receive hybrid prenatal care than urban residents.

Few nationally representative studies have explored temporal trends of rural vs urban and racial and ethnic disparities in hybrid prenatal care. Some studies using national self-reported survey data similarly reported that Black and Hispanic individuals were more likely to use telehealth compared with White individuals.^30,31,32^ However, results varied in studies using data from smaller geographic areas.^21,22^ For example, a study of 2 large academic nurse-midwifery clinics in Denver, Colorado, found lower uptake for prenatal telehealth care among Black and Hispanic patients compared with White patients.^22^ These data, either relying on self-report or conducted in 1 or 2 clinical sites, limited national generalizability and data on rural and urban differences.

During the COVID-19 PHE, CMS telehealth waivers and the CARES Act aimed to reduce policy-related barriers to telehealth implementation nationwide.^33^ Medicaid and commercial insurance plans also expanded access to telehealth by eliminating restrictions on the home as the originating site and by covering reproductive and maternal health services via telehealth.^34,35^ These policy measures led to a surge in telehealth prenatal care in the early months of the pandemic. However, many state Medicaid programs and commercial insurance plans have not permanently expanded all PHE telehealth flexibilities, including coverages of specific clinical services, audio-only services, and payment parity.^36^ This lack of universal telehealth flexibilities might explain the low uptake of prenatal telehealth services, with less than 6% of this study’s pregnant cohort using telehealth in combination with traditional in-person prenatal care. State- and plan-specific telehealth provisions will likely impact telehealth uptake and should be a focus of future studies.

Our finding that Black and Hispanic pregnant people had higher rates of prenatal care visits via telehealth than White individuals has multiple implications given the historical disparities in delayed or inadequate prenatal care for Black and Hispanic pregnant people.^22,37^ This shift in prenatal care modalities coincides with the first 9 months of the COVID-19 PHE (April-December 2020). During this period, stay-at-home orders were implemented in many states and pregnant individuals avoided visiting health care facilities due to fears of COVID-19. We found that Black and Hispanic people, especially those in urban communities, had a much larger increase in hybrid prenatal care than White individuals. Despite the increased percentage of Black and Hispanic individuals receiving hybrid prenatal care in our study, prior research indicated that overall prenatal care access did not measurably improve, as the proportion of Black individuals without care slightly increased from 3.3% to 3.5%, and from 2.5% to 2.7% for Hispanic individuals during 2019-2021.^2^ Addressing persistent disparities in prenatal care access through telehealth requires innovative solutions moving forward.

Policies that enable expansion of telehealth services for the prenatal period are likely to result in increased use across rural communities and could potentially be helpful in reducing current disparities in prenatal care access for individuals from minority communities.^10^ However, we found persistent rural and urban disparities in telehealth use, aligning with other research.^32^ Given the increasing hospital-based obstetric unit closures and maternal health workforce shortage in rural areas, expanding access to high-quality maternal care is particularly important in rural areas,^38,39^ potentially allowing for more timely and regular prenatal check-ins.^40^ Uneven distributions of prenatal telehealth uptake in rural vs urban areas may compel policymakers to develop necessary outreach efforts for rural residents to improve prenatal telehealth uptake, including the foci of broadband access and digital health literacy. In addition to patient-level interventions, recognizing health care professionals’ roles in the low uptake of prenatal telehealth care is critical. While patients may be willing to use telehealth, its implementation hinges primarily on health care services recommending, facilitating, and supporting its use. Many rural professionals may not promote telehealth or lack resources to help patients get connected and use these services effectively.^41,42^

### Limitations

This national retrospective cohort study has some limitations. First, telehealth services for prenatal care were identified using EHR data, which may have captured telehealth more frequently due to insurance reimbursement expansions and new coding guidance after March 2020, resulting in overestimated prepandemic and peripandemic differences in hybrid prenatal care. Second, this study used EHR data from geographically dispersed health systems, which allowed us to capture only the prenatal care services provided within these settings. Results might be different from those without prenatal care and/or childbirth care in these systems. Third, these EHR data do not include certain patient characteristics, which may provide key insight into the patterns of telehealth uptake, including health insurance information. Fourth, the N3C data were constructed for COVID-19 research; thus, the case-control design might result in an overrepresentation of sicker patients in this study, limiting our ability to generalize the findings to the national population. Fifth, the term hybrid prenatal care is not universally defined in perinatal health, and we defined it as combining in-person visits with telehealth for prenatal care. Due to intrinsic limitations of telehealth, such as inabilities to synchronously measure blood pressure and conduct laboratory tests, our results indicate that hybrid prenatal care was preferred over telehealth-only care, reflecting its integration into traditional in-person visits. Future work examining the comparative effectiveness of different hybrid models of care that use telehealth for triaging, routine, or follow-up care across trimesters is needed. Nevertheless, this study fills a knowledge gap in the large-scale nationwide prevalence of telehealth uptake for prenatal care over the recent 5 years and provides evidence on rural and urban and racial and ethnic differences in prenatal telehealth use. It would be useful to future research to examine the influence of telehealth or hybrid care on the timeliness of and overall access to care, quality (eg, adequacy of prenatal care), efficiency (eg, costs and number of prenatal care visits), and clinical effectiveness (eg, severe maternal morbidity and mortality), particularly among rural and racially and ethnically minority individuals.

## Conclusions

In this cohort study, prenatal telehealth care uptake significantly increased during the COVID-19 PHE yet remained used by few pregnant individuals during their prenatal period, suggesting the potential benefits of telehealth expansion are not fully realized. More importantly, rural individuals were less likely to use hybrid prenatal care, highlighting continual disproportionate access to telehealth among those who have historically faced barriers to prenatal care access. While the Centers for Disease Control and Prevention and American College of Obstetricians and Gynecologists have called for incorporating telehealth for average-risk pregnancies, our study highlights the urgent need to develop strategies that improve equitable access to telehealth for rural people and people in some minority racial and ethnic groups to address telehealth-associated health care disparities and optimize prenatal care across diverse populations.

## Notes

Supplement 1. **eFigure 1.** Temporal Pandemic Exposure Classifications of Prenatal Periods During the COVID-19 Public Health Emergency **eTable 1.** N3C Data, Key Study Variables and Concept Definitions **eTable 2.** List of Observational Medical Outcomes Partnership (OMOP) Common Data Model Concepts for Telehealth Services **eFigure 2.** Temporal Trends of Hybrid Prenatal Care and Prenatal Care Frequency among US Pregnant Individuals **eFigure 3.** Frequency of Pregnancies Initiating Prenatal Care and Prenatal Telehealth Visits by Gestational Week **eTable 3.** Maternal Characteristics of Study Cohort **eTable 4.** Distributions of Prenatal Care Utilization by Maternal Residence Rurality, Race/Ethnicity, and Age at Childbirth among Birthing People Giving Birth from June 2018 to May 2022 **eTable 5.** Differential Associations of Maternal Residence Location, Race/Ethnicity with Hybrid Prenatal Care by Prenatal Pandemic Exposure to the COVID-19 Pandemic **eTable 6.** Associations of Maternal Residence Location, Race/Ethnicity with Hybrid Prenatal Care among Pregnancies Fully Exposed to COVID-19 Public Health Emergencies during Prenatal Period

Supplement 2. **Nonauthor Collaborators.** National COVID Cohort Collaborative Consortium

Supplement 3.
Data Sharing Statement
