# Error in Author Name

**DOI:** 10.1001/jamanetworkopen.2024.59397

**Published:** 2025-01-10

In the Original Investigation titled “Racial and Ethnic and Rural Variations in the Use of Hybrid Prenatal Care in the US,”^1^ published December 6, 2024, there was a spelling error in an author name. The spelling of the ninth author “Nansi S. Boghoossian, PhD” should have been “Nansi S. Boghossian, PhD.” This article was corrected.^1^
