# Supplementary material for: Racial and Ethnic and Rural Variations in the Use of Hybrid Prenatal Care in the US
Source: JAMA Netw Open. 2024 Dec 6;7(12):e2449243. doi: 10.1001/jamanetworkopen.2024.49243 (PMC11624583; doi:10.1001/jamanetworkopen.2024.49243)
Supplement: Supplement 1. — eFigure 1. Temporal Pandemic Exposure Classifications of Prenatal Periods During the COVID-19 Public Health Emergency eTable 1. N3C Data, Key Study Variables and Concept Definitions eTable 2. List of Observational Medical Outcomes Partnership (OMOP) Common Data Model Concepts for Telehealth Services eFigure 2. Temporal Trends of Hybrid Prenatal Care and Prenatal Care Frequency among US Pregnant Individuals eFigure 3. Frequency of Pregnancies Initiating Prenatal Care and Prenatal Telehealth Visits by Gestational Week eTable 3. Maternal Characteristics of Study Cohort eTable 4. Distributions of Prenatal Care Utilization by Maternal Residence Rurality, Race/Ethnicity, and Age at Childbirth among Birthing People Giving Birth from June 2018 to May 2022 eTable 5. Differential Associations of Maternal Residence Location, Race/Ethnicity with Hybrid Prenatal Care by Prenatal Pandemic Exposure to the COVID-19 Pandemic eTable 6. Associations of Maternal Residence Location, Race/Ethnicity with Hybrid Prenatal Care among Pregnancies Fully Exposed to COVID-19 Public Health Emergencies during Prenatal Period [file jamanetwopen-e2449243-s001.pdf]

Supplementary Online Content

Hung P, Yu J, Harrison S, et al; National COVID Cohort Collaborative Consortium. Racial and ethnic and rural variations in the use of hybrid prenatal care in the US. *JAMA Netw Open*. 2024;7(12):e2449243. doi:10.1001/jamanetworkopen.2024.49243

eFigure 1. Temporal Pandemic Exposure Classifications of Prenatal Periods During the COVID-19 Public Health Emergency

eTable 1. N3C Data, Key Study Variables and Concept Definitions

eTable 2. List of Observational Medical Outcomes Partnership (OMOP) Common Data Model Concepts for Telehealth Services

eFigure 2. Temporal Trends of Hybrid Prenatal Care and Prenatal Care Frequency among US Pregnant Individuals

eFigure 3. Frequency of Pregnancies Initiating Prenatal Care and Prenatal Telehealth Visits by Gestational Week

eTable 3. Maternal Characteristics of Study Cohort

eTable 4. Distributions of Prenatal Care Utilization by Maternal Residence Rurality, Race/Ethnicity, and Age at Childbirth among Birthing People Giving Birth from June 2018 to May 2022

eTable 5. Differential Associations of Maternal Residence Location, Race/Ethnicity with Hybrid Prenatal Care by Prenatal Pandemic Exposure to the COVID-19 Pandemic

eTable 6. Associations of Maternal Residence Location, Race/Ethnicity with Hybrid Prenatal Care among Pregnancies Fully Exposed to COVID-19 Public Health Emergencies during Prenatal Period

This supplementary material has been provided by the authors to give readers additional information about their work.

© 2024 Hung P et al. *JAMA Network Open*.

eFigure 1. Temporal Pandemic Exposure Classifications of Prenatal Periods During the COVID-19 Public Health Emergency

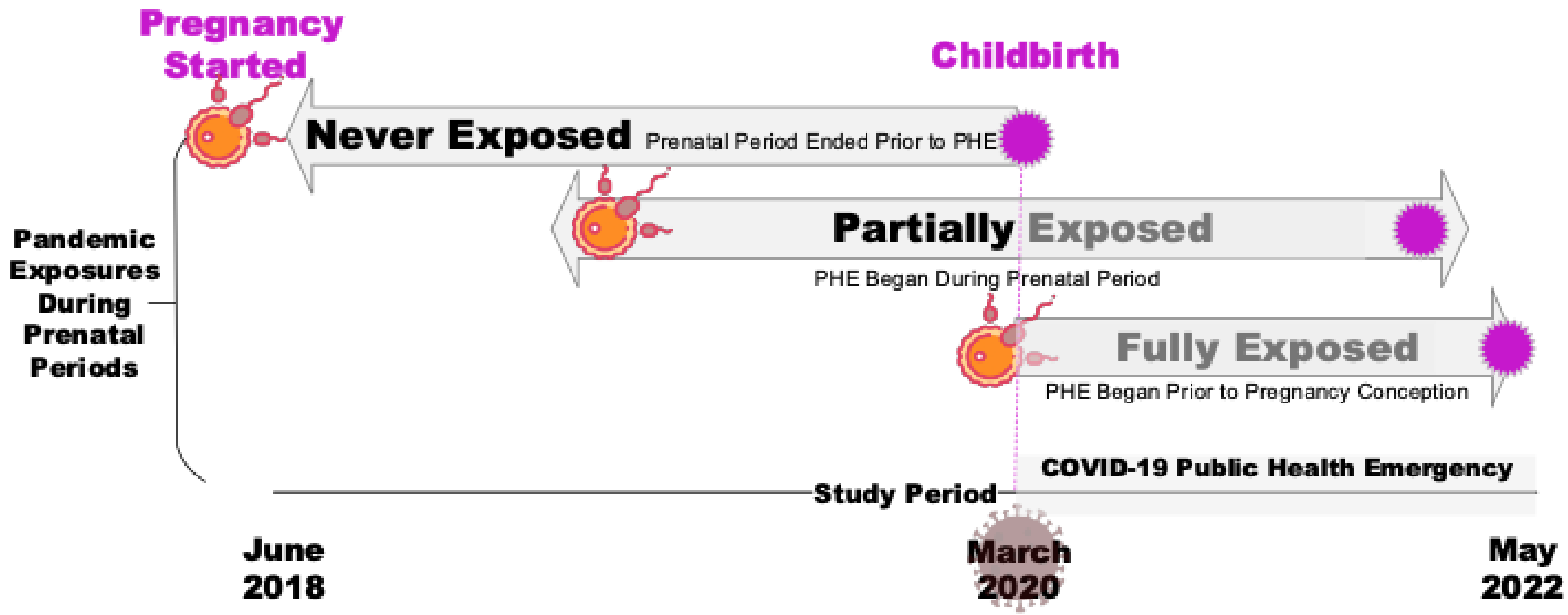

Notes: COVID-19 Public Health Emergency (PHE) occurred from March 1, 2020 to March 11, 2023.

© 2024 Hung P et al. *JAMA Network Open*.

eTable 1. N3C Data, Key Study Variables and Concept Definitions

The Phenotype & Data Acquisition workstream and contributing sites work collaboratively on data ingestion and harmonization in the N3C Enclave. Data payloads are updated approximately once a week by contributing sites. In partnership with each common data model (CDM) community, N3C harmonizes site data into Observational Medical Outcomes Partnership (OMOP) version 5.3.1. In the OMOP CDM, classification coding systems, such as International Classification of Diseases, Tenth Revision, Clinical Modification (ICD-10-CM), Current Procedural Terminology codes, Diagnosis Related Group (DRG) codes, Place of Services, Major Diagnostic Categories codes, and Standard Nomenclature of Medicine, are mapped to standard OMOP concepts based on semantic and clinical relationships, including laboratory measurements, clinical observations such as vital signs, medications, and clinical conditions. Vocabulary classification and mapping of various ontologies to the OMOP standard vocabulary is maintained by Observational Health Data Sciences and Informatics Network and publicly available on a web-based vocabulary repository, ATHENA.[27] We extracted records using these OMOP CDM. For prenatal records, we limited records to those that took place from an estimated pregnancy conception date to childbirth date to avoid inclusions of prenatal or intrapartum records from bundled claims.

**Variables**	Data Domain and OMOP CDM Concepts if Appropriate
Childbirth or intrapartum records	Condition_occurrence: 443445, 4163851, 4014456, 45757166, 45757167, 45772082, 4094046, 45765500, 45765501, 45765502, 4014295, 4014454, 4014296, 4014455, 4015162, 441641, 4205240, 193277, 4061457, 4066112, 4061458, 45757118, 4015270, 4063163, 4063162, 4059751, 4069200, 4066292, 4101844, 4128845, 4200201, 4126390, 4122720, 4096383, 4125602, 4009879, 433260, 3174660, 4014720, 4118903, 4063160, 442069, 4065737, 45757174, 4216316, 4009589, 4086393, 4092289, 4272248, 443213, 4054968, 4088584, 4014719<br><br>Procedure_occurrence Domain: 4073422, 2784567, 2784568, 2784569, 2784570, 2784571, 2784572, 2784578, 2784564, 2784565, 2784566, 2110307, 2110308, 2110309, 2110319, 2110320, 2110321, 2110316, 2110323<br><br>Observation Domain: 4014291, 4145318, 38001485, 38001486, 38001487, 38001488, 38001491, 38001492, 4264823, 44793347, 40482735, 40483126, 42539267, 36713468, 40483521, 36713074, 36713465, 42539210, 40483084, 40483101, 4262313, 4250009, 4192676, 4212794, 4250010, 4217586, 37310369, 37310393, 37310404, 4216797, 4236293, 44813089, 44802498
Gestational age at childbirth	Condition Occurrence Domain: 4218813, 4142581, 4167685, 4032055, 45757176, 4029320, 35625971, 4327745, 4244438, 4224646, 4294259, 45757175, 4240362, 45763591, 3657563, 4239938, 4078393, 4252252, 4034340, 45763590, 42538969, 4181468, 44791171, 4245908, 4270513, 4313026, 4290009, 444067, 442769, 45773507, 444098, 435655, 443871, 45757118, 442355, 444267, 443874, 441678, 433864, 434484, 4326232, 444417, 4180111, 432430, 444023, 435640, 4336226, 4274955, 4185780, 4220085, 4181751, 4097608, 4277749, 4283690, 4178165, 44791170, 4248725, 4266517, 4174506, 4242241, 4337360, 762907, 4062558, 4322726, 44791172, 438543, 442558, 444461, 439922, 4051642, 4049621, 4197245, 4132434, 4195157

© 2024 Hung P et al. *JAMA Network Open*.

	Procedure Occurrence Domain: 2793352, 2825091, 2871670, 2898834, 40487413, 36713272, 2793351, 2898832, 2898833, 2807693, 2793350, 2832899, 2871669, 2879607, 40486918, 4202200, 4290406, 2110327, 2793349, 2884808, 2840219, 2852789, 2211747, 2211748, 2211753, 2211754, 2793348, 2879606, 2898831, 2840218, 40488298, 40480885, 43020954, 4087135, 2110326, 2793347, 21493908, 21493909, 2892608, 2892609, 2807692, 44790206, 21494044, 21494042<br><br>Measurement: 3032525, 3032291, 40483600, 3037974, 3031374, 36203523, 3050129, 3050402, 3034062, 3037993<br><br>Observation Domain: 4112238, 4113140, 36305399, 3030256, 3031648, 4113139, 36304648, 40757033, 44817054
Mode of Delivery (Cesarean Delivery)	Condition_occurrence Domain: 45757118<br>Procedure_occurrence: 2784564, 2784565, 2784566, 2110316, 2110317, 2110318
Prenatal Records	Condition_occurrence Domain: 435887, 4063160, 72374, 72378, 72696, 72700, 73265, 73282, 75608, 76491, 76750, 76756, 76772, 77059, 78218, 78817, 78818, 79896, 80159, 80463, 81923, 192376, 192385, 193273, 193825, 193831, 195019, 196486, 196757, 196759, 197346, 200468, 313272, 315327, 315884, 432386, 432977, 433540, 433545, 434097, 434435, 435021, 435024, 435880, 435883, 435885, 436768, 437062, 437334, 437341, 437620, 437688, 437931, 437946, 439380, 439656, 440466, 80205, 134760, 135370, 194158, 195064, 198260, 199925, 200524, 258554, 258564, 260212, 260841, 313023, 313590, 319138, 374748, 433029, 436519, 438869, 439139, 606063, 606076, 606077, 3168171, 3655840, 3662256, 3663236, 4006329, 4006449, 4029320, 4034340, 4040733, 441365, 4048152, 4048166, 4048282, 4048283, 4048292, 4048460, 4048601, 4048602, 4048607, 4048610, 4048923, 4052173, 441649, 4071068, 4071069, 4071076, 4071083, 4071198, 4071717, 4071721, 4071735, 4071736, 4071737, 4071741, 4071743, 4079694, 4079851, 4079855, 4079973, 4080885, 4080888, 4082319, 4084442, 4085344, 4097959, 4105262, 4146935, 4147117, 4149586, 4162557, 4166754, 441926, 4170972, 4171091, 4171096, 4171097, 4171100, 4171102, 4171106, 4171108, 4171123, 4171691, 4172864, 4173000, 4173001, 4173002, 4173179, 4173181, 4173197, 4173198, 4173332, 442051, 4174299, 4174303, 4187201, 4237624, 4240362, 4243494, 4251487, 4258491, 4261839, 4263343, 4270073, 4278842, 4282746, 4283942, 4287783, 4293473, 4300467, 4301414, 4306199, 4306765, 4313830, 4313892, 4321550, 4322190, 4322958, 4345797, 36680586, 36712668, 36715840, 36715841, 36716757, 37017557, 37017566, 37019087, 37110521, 37119155, 42536566, 42536569, 42536745, 42537677, 42872436, 42872437, 42872438, 43021073, 44792382, 444094, 4047564, 442071, 442085, 442421, 442440, 442441, 442442, 443329, 3655881, 4025180, 4028475, ., 4028480, 4028621, 4028626, 4028629, 4028631, 4034075, 4034080, 4059763, 4059899, 4060034, 4060098, 4060240, 4060245, 4060259, 4060264, 4060559, 4060676, 4060807, 4061157, 4061347, 4061530, 4061791, 4061847, 4061971, 4062118, 4062122, 4063172, 4063175, 4063297, 4064152, 4064172, 4064175, 4064178, 4064299, 4064560, 4064722, 4064723, 4064724, 4064835, 4129015, 4129691, 4147338, 4152442, 4152443, 4152444, 4152445, 4156891, 4181975, 4310443, 35609140,

	35609141, 35609142, 35609143, 37208877, 37208879, 37312479, 44793476, 44793499, 44805139, 44805140, 45763661, 45763663<br><br>Procedure_occurrence Domain: 4112701, 2211747, 2211748, 2211749, 2211750, 2211751, 2211752, 2211753, 2211754, 2211755, 2211756, 2211757, 2211758, 2211759, 2211760, 2211761, 2722250, 2211762, 2211763, 2211764, 4299735, 4327042, 4334808, 43020944, 4168545, 2110284, 44513795, 46273020, 4070024, 2110283, 2004825, 2004811, 46271899, 37016939, 4237338, 4061130, 4059697, 40492793, 4082672, 4060624, 4060626, 4231954, 4237339, 40492794, 45773218, 37396331, 42535558, 42535559, 40489798, 4197294, 3655237, 4014148, 4014149, 4014150, 4014151, 4014152, 4014433, 4014434, 4014435, 4015141, 4015142, 4015296, 4015297, 4015298, 4015299, 4015300, 4015301, 4015302, 4047845, 4060265, 4061534, 4061803, 4103871, 4141415, 4143115, 4201730, 4221720, 4250610, 4254365, 37016920, 44807850, 44808732, 44808979, 44808980, 44808981, 45763755, 4070453, 2514574, 4082749, 4233889, 4237497, 4237498, 4238529, 4254364, 4257894, 4341109, 37017962, 37109725, 37310838, 40481948, 44789441, 44810615, 44810617, 46272368, 46272582, 2108255, 40757103, 2314189<br><br>Measurement Domain: 4014319, 4014320, 4015294, 4060266, 4061131, 4078285, 4087235, 4121342, 4191703, 4207466, 35609139, 36713462, 36713463, 36713464, 37016744, 37016760, 37310338, 37310544, 37393201, 44783612, 43020943, 4099476, 607563, 1175142, 1617152, 1617363, 3000707, 3000974, 3001059, 3001105, 3002087, 3002209, 3003169, 3003202, 3004059, 3004147, 3004222, 3004667, 3004864, 3005286, 3005749, 3005813, 3005823, 3006244, 3006855, 3006991, 3007137, 3007259, 3007898, 3007958, 3008667, 3008683, 3008867, 3009034, 3009212, 3009300, 3009518, 3009833, 3010209, 3010292, 3010506, 3012126, 3012266, 3012497, 3013324, 3014733, 3014867, 3014962, 3015257, 3015368, 3015381, 3015394, 3015873, 3016233, 3016409, 3016630, 3016758, 3017237, 3017302, 3017819, 3017938, 3018492, 3019326, 3020279, 3020352, 3021173, 3021744, 3021866, 3022447, 3022965, 3024243, 3024861, 3025305, 3025511, 3025686, 3026289, 3026348, 3026885, 3027098, 3027177, 3027334, 3027629, 3027765, 3028358, 3028596, 3028868, 3028875, 3028905, 3029141, 3029167, 3029195, 3029318, 3029440, 3029485, 3029487, 3029494, 3029645, 3029734, 3029739, 3029761, 3029767, 3029787, 3029788, 3030037, 3030065, 3030089, 3030318, 3030331, 3030342, 3030356, 3030370, 3030616, 3030627, 3030639, 3030659, 3030660, 3030668, 3030753, 3030783, 3030909, 3030922, 3030936, 3030955, 3032628, 3032802, 3033215, 3033223, 3033521, 3033849, 3034383, 3035156, 3035546, 3036322, 3036601, 3036683, 3036844, 3036934, 3037077, 3037440, 3037494, 3038036, 3038165, 3038618, 3038929, 3039154, 3040492, 3041363, 3041397, 3042795, 3042815, 3042823, 3042840, 3043110, 3043150, 3043386, 3043412, 3043437, 3043689, 3043708, 3043984, 3044815, 3044823, 3044824, 3044849, 3044851, 3044875, 3045119, 3045145, 3045167, 3045412, 3045419, 3045457, 3045689, 3045709, 3045717, 3045731, 3045991, 3046038, 3046295, 3046576, 3046602, 3046628, 3046852, 3046871, 3046886, 3046890, 3046912, 3047160, 3047186, 3047212, 3048230, 3048596, 3048886, 3049229, 3049518, 3049557, 3050129, 3050402, 3050433, 3053322, 4158633, 21492888, 21494348, 35951714, 36031183, 36031301, 36031461, 36031466, 36032422, 36203525, 36203526, 36305522, 36769326, 37019109, 37020081, 37310412, 37310413, 37310414, 37310839,

© 2024 Hung P et al. *JAMA Network Open*.

	40759059, 40759089, 40762033, 40763213, 40763214, 40765088, 40765745, 43533989, 46235157, 46235160, 46236732, 46236733<br><br>Observation Domain: 506742, 506743, 4014147, 4014321, 4015137, 4015139, 4015140, 4015295, 4047564, 4059987, 4059988, 4059989, 4060097, 4060101, 4060239, 4060241, 4060242, 4060243, 4060244, 4060246, 4060247, 4060250, 4060251, 4060258, 4060262, 4060263, 4061154, 4061156, 4061424, 4061425, 4061426, 4061428, 4061429, 4061432, 4061435, 4061437, 4061521, 4061529, 4061532, 4061786, 4061787, 4061789, 4061793, 4061802, 4062361, 4063309, 4079835, 4081292, 4083415, 4084186, 4087243, 4088025, 4137020, 4147564, 4147941, 4170305, 4173786, 4196580, 4237327, 4237344, 4237348, 4248961, 4254205, 4254206, 4257036, 4257038, 4257039, 35607944, 35609130, 35609131, 35609132, 35609133, 35609134, 35621778, 35621779, 35622068, 35622069, 35622070, 36674902, 36685904, 36685910, 36713461, 36717636, 37310337, 37310339, 37310340, 37310407, 37310408, 37310736, 40483081, 40760197, 42537958, 42689524, 43530748, 43530904, 44789715, 44793477, 44793478, 44793498, 44793526, 44793527, 44793541, 44793542, 44793543, 44793562, 44793576, 44804252, 44804550, 44808158, 44809543, 44809759, 44811395, 46285187, 4087235, 4203722, 4307024, 4330581, 601800, 762059, 1001654, 1002089, 1175602, 1175892, 1988091, 1988726, 1989262, 2101830, 2106904, 2414360, 2615489, 2615490, 2615491, 2618150, 2618151, 2618152, 2618153, 2618155, 2719397, 2719406, 2720486, 2720500, 2720904, 2721745, 3015917, 3016787, 3024174, 3030256, 3030581, 3031648, 3031834, 3032496, 3033198, 3038892, 3038905, 3039742, 3040319, 3040441, 3040643, 3174513, 3657601, 3657602, 3657603, 3657604, 3657605, 3657606, 3657607, 3657608, 3657609, 3657610, 3657611, 3657612, 3657613, 3657614, 3657619, 3657620, 3657621, 3657622, 3657623, 3657624, 4024541, 4030301, 4030911, 4045503, 4050374, 4055255, 4085346, 4114006, 4122053, 4145062, 4155796, 4170112, 4170971, 4172038, 4175499, 4189869, 4196004, 4215846, 4224773, 4264822, 4295020, 4296382, 4305726, 4310193, 4311447, 4313474, 21491261, 21491267, 21491268, 21491269, 21491270, 21491271, 21491272, 21491273, 21492587, 21492594, 21492599, 21492607, 21492611, 21492618, 21492721, 21492733, 21492738, 21492742, 21492748, 21493000, 21493785, 21493812, 21495028, 35609156, 35609175, 36031190, 36031972, 36032080, 36032327, 36203290, 36303278, 36303279, 36303318, 36303430, 36303568, 36303597, 36303600, 36303619, 36303666, 36303791, 36304061, 36304126, 36304139, 36304169, 36304222, 36304304, 36304358, 36304421, 36304609, 36304788, 36304866, 36305127, 36305164, 36305625, 36305635, 36305648, 36305690, 36305815, 36305964, 36305968, 36306076, 36306137, 36306217, 37310822, 37310823, 37310836, 37396294, 38001655, 40218315, 40218323, 40759202, 40760190, 40760192, 40760298, 40760825, 40771078, 40771079, 40771140, 40771141, 40771142, 40771144, 40771145, 40771146, 40771147, 40771148, 40771149, 40771256, 40771257, 40771258, 40771259, 40771565, 42527102, 42527158, 42527187, 42527188, 42527395, 42527523, 42528335, 42528336, 42528453, 42528454, 42528455, 42528966, 42529441, 42536210, 42579191, 42587222, 42587223, 42587224, 42587225, 42590299, 42592120, 42628127, 42628256, 42628264, 42628270, 42628295, 42628317, 42628377, 43021798, 43054890, 43533800, 43533837, 44793003, 44794767, 44794814, 44797301, 44798416, 44798468, 44799057, 44799058, 44800038, 44800947, 44804720, 44805354, 44805365, 44808043, 44808516, 44808630, 44811893, 44813328, 44814170, 44817052, 44817053, 44817089, 44817093, 44817223, 45763686,

© 2024 Hung P et al. *JAMA Network Open*.

	45765514, 45765515, 45773223, 46234792, 46234841, 46235158, 46236884, 46270506, 46272536, 4219847, 4024607, 2101829, 2101831, 2108529, 2108689, 2109552<br><br>Device Domain: 45349212, 45383356, 45764427, 45764531
Telehealth	Procedure_occurrence Domain: 709752, 710056, 710057, 710058, 710060, 927187, 927192, 801014, 953288, 953289, 953290, 1314339, 1314340, 2101773, 2101774, 2314337, 2314338, 2314339, 2314340, 2414364, 2514548, 2514549, 2514550, 2514551, 36716324, 37396332, 40659402, 40663506, 40663507, 44786387, 45889008, 45889547<br><br>Observation Domain: 603231, 608470, 608471, 608473, 608474, 608475,608476, 618899, 618900, 618901, 618902, 618903, 44812167, 709799, 710061, 764939, 953388, 1175166, 1175171, 1175199,1175262, 1175297, 1175329, 1175357, 1175366, 1175425, 1175432, 1175572, 1175587, 1175656, 1175892, 1175897,1175899, 1175962, 1175975, 1176010, 1176034, 1176047, 1176054, 1176061, 1176141, 1176162, 1176411, 1176466, 1616354,1988345, 2617473, 2617474, 2617475, 2720700, 2721636, 36032129, 36032256, 36032260, 36033671, 36203708, 36203731,36303477, 36304778, 36306033, 36659703, 36659758, 36659787, 36659852, 36659858, 36659875, 36659877, 36659894,36659898, 36659946, 36659973, 36659984, 36659985, 36659998, 36660000, 36660022, 36660047, 36660051, 36660071,36660088, 36660098, 36660179, 36660214, 36660249, 36660267, 36660278, 36660286, 36660333, 36660339, 36660418,36660442, 36660453, 36660529, 36660535, 36660621, 36660628, 36660633, 36660654, 36660655, 36660658, 36660660,36660675, 36714563, 36714564, 36717109, 36717548, 37020540, 37021046, 37395677, 37395731, 37395732, 37396521,42527874, 42528003, 42528004, 42528085, 42528086, 42528087, 42528260, 42528261, 42528262, 42528304, 42529398,42628097, 42628109, 43021425, 43528032, 43528033, 44783192, 44803305, 44803306, 44810185, 44812159, 45773597, 45888153, 46235112, 46235113,46235114, 46285869,42628109<br>Visit_occurrence Domain: 5083<br>Device Domain: 2618090<br>Meas_value Domain: 706416, 1620319, 1621168, 46237365, 42628422, 36309282, 45880527
Overweight/obesity	Condition Occurrence Domain: 433736, 4217557, 434005,4097996, 4100857, 4029276, 4029900, 4235799, 4097929, 4182506, 4160821, 4005991, 4215969, 4081038, 37204685, 37204815, 3199162, 36715355, 37206117, 37397209, 36716151, 42872398, 37204691, 4183240, 36716555, 45757112, 4177337, 4220527, 4203289, 4171317, 37018860, 4087487, 37208175, 37311904, 4093860, 4176962, 4184965, 45766204, 44805740, 44805739, 44805738, 44805741, 4212443, 4189665, 4185912, 4163032, 4171147, 42539192<br><br>Observation: 4060985, 4256640, 437525, 4060705, 44811711, 4215968, 763589, 37311728, 44811712, 35622268, 4152039, 4063241, 4061918, 44809878, 4189664<br><br>Note: 46234997, 36031198, 1175923, 1176203, 1176401
Smoking during Pregnancy	Condition Occurrence Domain: 437264, 4099811, 37109024, 4317149, 4103417, 4103418, 44799960, 4315821, 764468, 764471, 37109023, 4176241,

© 2024 Hung P et al. *JAMA Network Open*.

	36716473, 36716478, 37203948, 765451, 764469, 37109023, 4263877, 440612, 4209423<br><br>Observation: 4141787, 764104, 762498, 764103, 4144273, 4218917, 4052030, 44789712, 4044777, 4209585, 4052948, 37395605, 4041511, 762499, 4052029, 4215409, 4046886, 4058136, 4058137, 4276526, 4275495, 4132133, 4282779, 4209006, 4216174, 4190573, 4044778, 4058138, 4298794, 4042037, 4052947, 40486518, 4269997, 4141786, 4044776, 42709996, 4044775, 4246415, 4204653, 4144271, 4041508, 4041509, 44809281, 4047454, 4269183, 36684331
Preexisting/Gestational Diabetes	Condition Occurrence Domain: 4058243, 4063042, 43531008, 45757079, 4063043, 43531010, 43531007, 4024659, 45757124, 45757789, 45757129, 195771, 4226238, 443727, 4009303, 4095288, 442793, 192279, 443767, 4174977, 380097, 378743, 35626038, 35626039, 37016358, 35626041, 35626042, 376114, 4290822, 35626043, 35626044, 4266637, 380096, 4255401, 200687, 42538169, 4227210, 37016179, 37016180, 45763583, 45763584, 45769873, 4225656, 377821, 4225055, 37017431, 37016767, 4143857, 318712, 4223303, 435216, 4152858, 45769830, 45769832, 45770902, 4099214, 45769876, 4228112, 37016348, 4196141, 43531563, 43531616, 43530690, 4099651, 36714116, 45757363, 4338901, 4008576, 443412, 201826, 443735, 201530, 443734, 4228443, 443731, 43531578, 443733, 45770830, 45757435, 45770881, 43530656, 43530685, 45773064, 4221495, 376065, 4222415, 37017432, 37016768, 4140466, 443729, 4222876, 443732, 443730, 4044391, 4048028, 376112, 40484648, 40482801, 4252356, 4338896, 35626068, 4215961, 4175440, 4191611, 321822, 4131908, 4226354, 46270483, 4114427, 4033942, 4159742, 4227657, 4029422, 4226798, 4029423, 4029420, 201254, 439770, 376979, 201531, 443592, 194700, 4202383, 37016356, 37016350, 35626067, 4224254, 37110593, 37016349, 201820, 377552, 443238, 4338897, 4212441<br><br>Observation Domain: 4016041, 4016041, 4016042
Preexisting and pregnancy-induced Hypertension	Condition Occurrence Domain: 320128, 317898, 312648, 442604, 4183981, 314369, 316994, 4263504, 313502, 439698, 319034, 193493, 312938, 442626, 195556, 319826, 318437, 4110947, 314958, 4249016, 317895, 321638, 197930, 321074, 141084, 439694, 444101, 443919, 439696, 4062552, 45757356, 4057978, 45757139, 45757137, 4057979, 44784439, 201313, 44782690, 44784621, 314378, 44782429, 442766, 43021852, 43020455, 439695, 4283352, 42538946, 4311246, 44782728, 442603
Depression and Anxiety During Pregnancy	Condition Occurrence Domain: 4282096, 4282316, 440383, 44784632, 432866, 432290, 440067, 4030856, 4220617, 4327669, 4148842, 437249, 443237, 438119, 439272, 438405, 439262, 437831, 4195572, 4049623, 435220, 4263748, 4012108, 440078, 441834, 433992, 443797, 439256, 439255, 439254, 439253, 437528, 442570, 436386, 439251, 443906, 439249, 439248, 372599, 439246, 437529, 4211231, 434911, 433991, 4011623 432883, 4147466, 4219627, 440690, 4336957, 4307111, 4327337, 4250023, 4323418, 4098302, 4228802, 4077577, 4141454, 432285, 4321835, 4189538, 4058397, 4239656, 43020470, 4085062, 434613, 4304010, 440985, 438998, 4268775, 4182847, 4184002, 4085678, 4012102, 4025952, 4009714, 4092616, 44783317, 43020471, 439245, 432876, 436665, 440980, 438727, 435520, 4113821, 441534, 438406, 4025677

© 2024 Hung P et al. *JAMA Network Open*.

Plurality	Condition Occurrence Domain: 4014296, 4014456, 4163851, 4014455, 45757166, 45757167, 45772082, 4094046, 45765500, 45765501, 45765502, 432969, 441919, 43021952, 435357, 43021951, 43021054, 432375, 442594, 4063158, 4129021, 4330861, 444386, 4249470, 4049777, 4252093, 4110123, 4289742, 4147942, 4328361, 442422, 4015162, 72700, 4234847, 4187796, 4216512, 45770043, 42538805, 45757168, 45770044, 42539377, 45773428, 37017323, 435018, 434097, 4118413, 43021057, 43021056, 3185356, 3662136, 3662135, 3657367, 3662133, 3657368, 3662134, 440462, 437931, 4118398, 4126072, 4178154, 442082, 4145125, 4016474, 45757769, 4185557, 438481, 440466, 45757730, 4015163, 4015422, 4015421, 45770042, 45757165, 42535052, 42538806, 45757169, 37017322, 43021055, 4118411, 44806034, 4276503, 4319561, 4101844<br><br>Procedure: 2793348, 2793350, 2793352, 21493907, 21493908, 2879606, 2832899, 2825091, 21493910, 21493909, 44807918, 44807919, 40492794, 4196949, 40486662, 4206892, 4107573, 44515960<br>Observation Domain: 36717637, 36713469, 45769106, 3045823, 4226978, 36716289, 40483101, 40483084, 36713468, 4272815, 1616913
SARS-CoV-2 Infection	Measurement: 586525, 36032174, 36032174, 723472, 706155, 723468, 36661370, 723467, 586520, 723465, 586516, 706167, 723477, 715262, 706172, 715260, 586519, 723469, 586529, 586523, 36031506, 723466, 723476, 36032258, 586526, 586518, 36031213, 757677, 706154, 706158, 706161, 723470, 36031652, 36661378, 706159, 706174, 706156, 586528, 37310257, 706166, 36032419, 706169, 36031238, 706160, 715272, 757678, 715261, 706170, 706157, 723478, 706171, 757685, 36661377, 36661371, 723471, 36031453, 706173, 706175, 706168, 586524, 723464, 706165, 36032061, 706163, 36031944, 723463, 706181,723459, 706180, 36659631, 36661373, 757680, 723479, 706176, 36661374, 706178, 723473, 757679, 586515, 706177, 36031734, 36661372, 586522, 757686, 37310258, 723474, 706179, 36031969, 36032309, 586521, 36031197, 36661369, 723475, 723480, 586527, 36031956<br><br>Meas Value: 36715206, 9191, 36032716, 45884084, 45877985, 45878745, 45881802, 4126681<br><br>Condition: 37311061
Urban/Rural Residence	Person<br>Location
Residence Census Region	Person<br>Location

© 2024 Hung P et al. *JAMA Network Open*.

eTable 2. List of Observational Medical Outcomes Partnership (OMOP) Common Data Model Concepts for Telehealth Services

Concept ID	Code	Name	Standard Class	Domain	Vocab
5083	02	Telehealth	Visit	Visit	CMS Place of Service
2618090	GQ	Via asynchronous telecommunications system	HCPCS Modifier	Device	HCPCS
706416	LP173058-1	Telehealth	Doc Setting	Meas Value	LOINC
1620319	LA31380-1	Phone-based telehealth	Answer	Meas Value	LOINC
1621168	LA31382-7	Video-based telehealth	Answer	Meas Value	LOINC
36309282	LA28023-2	Obtained change in care plan orders (for example increased monitoring by agency; change in visit frequency; telehealth)	Answer	Meas Value	LOINC
42628422	GQ	Via asynchronous telecommunications system	CPT4 Modifier	Meas Value	CPT4
45880527	LA12242-6	Obtained change in care plan orders (for example telephone)	Answer	Meas Value	LOINC
46237365	LA24009-5	Synchronous communication (e.g. telephone or telehealth video visit)	Answer	Meas Value	LOINC
603231	1325261000000100	Referral by telehealth pulse oximetry monitoring service	Procedure	Observation	SNOMED
608470	1325191000000100	Telehealth pulse oximetry monitoring started	Context-dependent	Observation	SNOMED
608471	1325201000000100	Telehealth pulse oximetry monitoring ended	Context-dependent	Observation	SNOMED
608473	1325221000000100	Telehealth pulse oximetry monitoring not appropriate	Clinical Finding	Observation	SNOMED
608475	1325251000000100	Referral to telehealth pulse oximetry monitoring service	Procedure	Observation	SNOMED
608476	1325271000000100	Discharge from telehealth pulse oximetry monitoring service	Procedure	Observation	SNOMED
618899	879780004	Telehealth monitoring for chronic heart failure	Procedure	Observation	SNOMED
618900	879781000	Telehealth monitoring for ischemic heart disease	Procedure	Observation	SNOMED
618901	879782007	Telehealth monitoring for diabetes mellitus	Procedure	Observation	SNOMED
618902	879783002	Telehealth monitoring for psychological stress	Procedure	Observation	SNOMED
618903	879784008	Telehealth rehabilitation monitoring	Procedure	Observation	SNOMED
710061	99474	Self-measured blood pressure using a device validated for clinical accuracy; separate self-measurements of two readings one minute apart, twice daily over a 30-day period (minimum of 12 readings), collection of data reported by the patient and/or caregiver	CPT4	Observation	CPT4
764939	8861000175100	Telehealth service	Qualifier Value	Observation	SNOMED
953388	G0	Telehealth services for diagnosis	HCPCS Modifier	Observation	HCPCS
1175166	90714-7	Pain medicine Telehealth Consult note	Clinical Observation	Observation	LOINC

© 2024 Hung P et al. *JAMA Network Open*.

Concept ID	Code	Name	Standard Class	Domain	Vocab
1175171	90008-4	Pharmacogenomics Telehealth Note	Clinical Observation	Observation	LOINC
1175199	89901-3	Sports medicine Telehealth Note	Clinical Observation	Observation	LOINC
1175262	89886-6	Palliative care Telehealth Note	Clinical Observation	Observation	LOINC
1175297	89887-4	Adolescent medicine Telehealth Note	Clinical Observation	Observation	LOINC
1175329	89889-0	Physical medicine and rehab Telehealth Note	Clinical Observation	Observation	LOINC
1175357	89884-1	Hematology+Medical oncology Telehealth Note	Clinical Observation	Observation	LOINC
1175366	89891-6	Endocrinology Telehealth Note	Clinical Observation	Observation	LOINC
1175425	89902-1	Otolaryngology Telehealth Note	Clinical Observation	Observation	LOINC
1175432	90713-9	Sleep medicine Telehealth Note	Clinical Observation	Observation	LOINC
1175572	89888-2	Pediatrics Telehealth Note	Clinical Observation	Observation	LOINC
1175587	89899-9	Orthopaedic surgery Telehealth Note	Clinical Observation	Observation	LOINC
1175656	89898-1	Nephrology Telehealth Note	Clinical Observation	Observation	LOINC
1175892	89894-0	Neonatal perinatal medicine Telehealth Note	Clinical Observation	Observation	LOINC
1175897	89893-2	Clinical genetics Telehealth Note	Clinical Observation	Observation	LOINC
1175899	89883-3	Emergency medicine Telehealth Note	Clinical Observation	Observation	LOINC
1175962	89897-3	Gynecology Telehealth Note	Clinical Observation	Observation	LOINC
1175975	89892-4	Infectious disease Telehealth Note	Clinical Observation	Observation	LOINC
1176010	89890-8	Diabetology Telehealth Note	Clinical Observation	Observation	LOINC
1176034	89896-5	Developmental-behavioral pediatrics Telehealth Note	Clinical Observation	Observation	LOINC

© 2024 Hung P et al. *JAMA Network Open*.

Concept ID	Code	Name	Standard Class	Domain	Vocab
1176047	90715-4	Palliative care Telehealth Consult note	Clinical Observation	Observation	LOINC
1176054	89903-9	Urology Telehealth Note	Clinical Observation	Observation	LOINC
1176061	89900-5	Rheumatology Telehealth Note	Clinical Observation	Observation	LOINC
1176141	89895-7	Allergy and Immunology Telehealth Note	Clinical Observation	Observation	LOINC
1176162	89885-8	Pain medicine Telehealth Note	Clinical Observation	Observation	LOINC
1176411	90712-1	Sleep medicine Telehealth Consult note	Clinical Observation	Observation	LOINC
1176466	90716-2	Psychiatry Telehealth Note	Clinical Observation	Observation	LOINC
1616354	97569-8	Eating disorders Telehealth Note	Clinical Observation	Observation	LOINC
1988345	99479-8	Physical therapy Telehealth Note	Clinical Observation	Observation	LOINC
2617473	G0406	Follow-up inpatient consultation physicians typically spend 15 minutes communicating with the patient via telehealth	HCPCS	Observation	HCPCS
2617474	G0407	Follow-up inpatient consultation physicians typically spend 25 minutes communicating with the patient via telehealth	HCPCS	Observation	HCPCS
2617475	G0408	Follow-up inpatient consultation physicians typically spend 35 minutes communicating with the patient via telehealth	HCPCS	Observation	HCPCS
2720700	Q3014	Telehealth originating site facility fee	HCPCS	Observation	HCPCS
2721636	T1014	Telehealth transmission per minute professional services bill separately	HCPCS	Observation	HCPCS
36032129	95520-3	Integrative medicine Telehealth Note	Clinical Observation	Observation	LOINC
36032256	95804-1	Breastfeeding Telehealth Note	Clinical Observation	Observation	LOINC
36032260	96332-2	Case manager Telehealth Note	Clinical Observation	Observation	LOINC
36033671	96739-8	Telehealth COVID-19 note	Clinical Observation	Observation	LOINC
36203708	85888-6	Multi-specialty program Telehealth Note	Clinical Observation	Observation	LOINC

© 2024 Hung P et al. *JAMA Network Open*.

Concept ID	Code	Name	Standard Class	Domain	Vocab
36203731	86208-6	Cardiology Telehealth Note	Clinical Observation	Observation	LOINC
36303477	89033-5	Urology Telehealth Consult note	Clinical Observation	Observation	LOINC
36304778	89032-7	Nephrology Telehealth Consult note	Clinical Observation	Observation	LOINC
36306033	89031-9	Hematology Telehealth Consult note	Clinical Observation	Observation	LOINC
36659703	94788-7	Clinical pathology Telehealth Note	Clinical Observation	Observation	LOINC
36659758	94775-4	Critical care medicine Telehealth Note	Clinical Observation	Observation	LOINC
36659787	94725-9	Aerodigestive medicine Telehealth Note	Clinical Observation	Observation	LOINC
36659852	94778-8	Ethics Telehealth Note	Clinical Observation	Observation	LOINC
36659858	94724-2	Pulmonary Telehealth Note	Clinical Observation	Observation	LOINC
36659875	94802-6	Respiratory therapy Telehealth Note	Clinical Observation	Observation	LOINC
36659877	94790-3	Nutrition and dietetics Telehealth Note	Clinical Observation	Observation	LOINC
36659894	94770-5	Wound and Continence Care Telehealth Note	Clinical Observation	Observation	LOINC
36659898	94789-5	Maternal and fetal medicine Telehealth Note	Clinical Observation	Observation	LOINC
36659946	94780-4	Gastroenterology Telehealth Note	Clinical Observation	Observation	LOINC
36659973	94784-6	Gynecologic oncology Telehealth Note	Clinical Observation	Observation	LOINC
36659984	94804-2	Trauma Telehealth Note	Clinical Observation	Observation	LOINC
36659985	94776-2	Dentistry Telehealth Note	Clinical Observation	Observation	LOINC
36659998	94787-9	Interventional radiology Telehealth Note	Clinical Observation	Observation	LOINC
36660000	94774-7	Cardiac surgery Telehealth Note	Clinical Observation	Observation	LOINC

© 2024 Hung P et al. *JAMA Network Open*.

Concept ID	Code	Name	Standard Class	Domain	Vocab
36660022	94781-2	General medicine Telehealth Note	Clinical Observation	Observation	LOINC
36660047	94771-3	Anesthesiology Telehealth Note	Clinical Observation	Observation	LOINC
36660051	94793-7	Wound care management Telehealth Note	Clinical Observation	Observation	LOINC
36660071	94777-0	Dermatology Telehealth Note	Clinical Observation	Observation	LOINC
36660088	94792-9	Oncology Telehealth Note	Clinical Observation	Observation	LOINC
36660098	94594-9	Neurological surgery Telehealth Note	Clinical Observation	Observation	LOINC
36660179	94773-9	Burn management Telehealth Note	Clinical Observation	Observation	LOINC
36660214	94779-6	Family medicine Telehealth Note	Clinical Observation	Observation	LOINC
36660249	94782-0	Surgery Telehealth Note	Clinical Observation	Observation	LOINC
36660267	94595-6	Pharmacology Telehealth Medication management note	Clinical Observation	Observation	LOINC
36660278	94794-5	Ophthalmology Telehealth Note	Clinical Observation	Observation	LOINC
36660286	94726-7	Cleft and Craniofacial Telehealth Note	Clinical Observation	Observation	LOINC
36660333	94797-8	Plastic surgery Telehealth Note	Clinical Observation	Observation	LOINC
36660339	94795-2	Pastoral care Telehealth Note	Clinical Observation	Observation	LOINC
36660418	94772-1	Blood banking and transfusion medicine Telehealth Note	Clinical Observation	Observation	LOINC
36660442	94785-3	Healthcare navigator Telehealth Note	Clinical Observation	Observation	LOINC
36660453	94803-4	Speech-language pathology Telehealth Note	Clinical Observation	Observation	LOINC
36660529	94786-1	Hematology Telehealth Note	Clinical Observation	Observation	LOINC
36660535	94791-1	Occupational therapy Telehealth Note	Clinical Observation	Observation	LOINC

© 2024 Hung P et al. *JAMA Network Open*.

Concept ID	Code	Name	Standard Class	Domain	Vocab
36660621	94783-8	Geriatric medicine Telehealth Note	Clinical Observation	Observation	LOINC
36660628	94801-8	Recreational therapy Telehealth Note	Clinical Observation	Observation	LOINC
36660633	94798-6	Psychology Telehealth Note	Clinical Observation	Observation	LOINC
36660654	95407-3	Cardiopulmonary Telehealth Note	Clinical Observation	Observation	LOINC
36660655	94799-4	Radiation oncology Telehealth Note	Clinical Observation	Observation	LOINC
36660658	94800-0	Rapid response team Telehealth Note	Clinical Observation	Observation	LOINC
36660660	94805-9	Vascular surgery Telehealth Note	Clinical Observation	Observation	LOINC
36660675	94796-0	Pharmacology Telehealth Note	Clinical Observation	Observation	LOINC
36714563	719856008	Suitable for telehealth monitoring	Clinical Finding	Observation	SNOMED
36714564	719858009	Telehealth monitoring	Procedure	Observation	SNOMED
36717109	719857004	Telehealth monitoring suspended	Context-dependent	Observation	SNOMED
36717548	722299009	Step up change in telehealth monitoring	Procedure	Observation	SNOMED
37020540	91531-4	Social worker Telehealth Note	Clinical Observation	Observation	LOINC
37021046	92913-3	Immunology Telehealth Note	Clinical Observation	Observation	LOINC
37395677	715191006	Telehealth asthma monitoring	Procedure	Observation	SNOMED
37395731	715279006	Telehealth obesity monitoring	Procedure	Observation	SNOMED
37395732	715280009	Telehealth hypertension monitoring	Procedure	Observation	SNOMED
37396521	716358000	Telehealth chronic obstructive pulmonary disease monitoring	Procedure	Observation	SNOMED
42527874	84009-0	Spinal cord injury medicine Telehealth Note	Clinical Observation	Observation	LOINC
42528003	84069-4	Multi-specialty program Telehealth Summary note	Clinical Observation	Observation	LOINC
42528004	84070-2	Multi-specialty program Telehealth Plan of care note	Clinical Observation	Observation	LOINC
42528085	85206-1	Telehealth Initial evaluation note	Clinical Observation	Observation	LOINC

© 2024 Hung P et al. *JAMA Network Open*.

Concept ID	Code	Name	Standard Class	Domain	Vocab
42528086	85207-9	Telehealth Education note	Clinical Observation	Observation	LOINC
42528087	85208-7	Telehealth Consult note	Clinical Observation	Observation	LOINC
42528260	84231-0	Neurology Telehealth Consult note	Clinical Observation	Observation	LOINC
42528261	84232-8	Neurology Telehealth Note	Clinical Observation	Observation	LOINC
42528262	84233-6	Neurology Telehealth Initial evaluation note	Clinical Observation	Observation	LOINC
42528304	84274-0	Nurse Telehealth Note	Clinical Observation	Observation	LOINC
42529398	84215-3	Mental health Telehealth Note	Clinical Observation	Observation	LOINC
42628097	95	Synchronous telemedicine service rendered via a real-time interactive audio and video telecommunications system	CPT4 Modifier	Observation	CPT4
42628109	GT	Via interactive audio and video telecommunication systems	CPT4 Modifier	Observation	CPT4
43021425	473199000	Telehealth monitoring for chronic disease	Procedure	Observation	SNOMED
43528032	99495	Transitional Care Management Services with the following required elements: Communication (direct contact, telephone, electronic) with the patient and/or caregiver within 2 business days of discharge Medical decision making of at least moderate complex...	CPT4	Observation	CPT4
43528033	99496	Transitional Care Management Services with the following required elements: Communication (direct contact, telephone, electronic) with the patient and/or caregiver within 2 business days of discharge Medical decision making of high complexity during th...	CPT4	Observation	CPT4
44783192	699249000	Alert received from telehealth monitoring system	Context-dependent	Observation	SNOMED
44803305	726861000000100	Starting of telehealth monitoring	Procedure	Observation	SNOMED
44803306	726871000000107	Ending of telehealth monitoring	Procedure	Observation	SNOMED
44810185	886611000000107	Telehealth monitoring (patient participation not appropriate)	Clinical Finding	Observation	SNOMED
44812159	911231000000103	Telehealth monitoring service	Qualifier Value	Observation	SNOMED
44812167	911381000000108	Telehealthcare service	Qualifier Value	Observation	SNOMED
45773597	922451000000105	Telehealth monitoring invitation	Procedure	Observation	SNOMED
46235112	75496-0	Telehealth Note	Clinical Observation	Observation	LOINC

© 2024 Hung P et al. *JAMA Network Open*.

Concept ID	Code	Name	Standard Class	Domain	Vocab
46235113	75497-8	Telehealth Progress note	Clinical Observation	Observation	LOINC
46235114	75498-6	Telehealth Summary note	Clinical Observation	Observation	LOINC
46285869	977531000000104	Referral to telehealth service	Procedure	Observation	SNOMED
709752	G0	Telehealth services for diagnosis	CPT4 Modifier	Procedure	CPT4
710056	99421	Non-Face-to-Face On-Line Digital E&M Service, 5-10 mins	Procedure	Procedure	CPT4
710057	99422	Non-Face-to-Face On-Line Digital E&M Service, 11-20 mins	Procedure	Procedure	CPT4
710058	99423	Non-Face-to-Face On-Line Digital E&M Service, 21+ mins	Procedure	Procedure	CPT4
710060	99473	Self-measured blood pressure using a device validated for clinical accuracy; patient education/training and device calibration	CPT4	Procedure	CPT4
801014	G2025	RHC/FQHC distant site telehealth service	HCPCS	Procedure	HCPCS
927187	99452	Interprofessional telephone/Internet/electronic health record referral service(s) provided by a treating/requesting physician or other qualified health care professional, 30 minutes	CPT4	Procedure	CPT4
927192	99451	Interprofessional telephone/Internet/electronic health record assessment and management service provided by a consultative physician, including a written report to the patient's treating/requesting physician or other qualified health care professional,...	CPT4	Procedure	CPT4
953288	G2012	Brief communication technology-based service, e.g. virtual check-in, to an established patient	HCPCS	Procedure	HCPCS
953289	G2010	Remote evaluation of recorded video and/or images submitted by an established patient	HCPCS	Procedure	HCPCS
953290	G0071	Payment for communication technology-based services for 5 minutes or more of a virtual (non-face-to-face) communication between an rural health clinic (rhc) or federally qualified health center (fqhc) practitioner and rhc or fqhc patient, or 5 minutes or more of remote evaluation of recorded video and/or images by an rhc or fqhc practitioner, occurring in lieu of an office visit; rhc or fqhc only	HCPCS	Procedure	HCPCS
1314339	G0508	Telehealth consultation physicians typically spend 60 minutes communicating with the patient and providers via telehealth	HCPCS	Procedure	HCPCS
1314340	G0509	Telehealth consultation physicians typically spend 50 minutes communicating with the patient and providers via telehealth	HCPCS	Procedure	HCPCS
2101773	0188T	Remote real-time videoconference critical care	CPT4	Procedure	CPT4
2101774	0189T	Remote real-time videoconference critical care	CPT4	Procedure	CPT4

© 2024 Hung P et al. *JAMA Network Open*.

Concept ID	Code	Name	Standard Class	Domain	Vocab
2314337	98966	A nonphysician provider telephone E&M services, 5-10 mins	Procedure	Procedure	CPT4
2314338	98967	A nonphysician provider telephone E&M services, 11-20 mins	Procedure	Procedure	CPT4
2314339	98968	A nonphysician provider telephone E&M services, 21-30 mins	Procedure	Procedure	CPT4
2314340	98969	Brief check-in or e-visit	Procedure	Procedure	CPT4
2414364	99091	Collection and interpretation of physiologic data (eg, ECG, blood pressure, glucose monitoring) digitally stored and/or transmitted by the patient and/or caregiver to the physician or other qualified health care professional, qualified by education, tr...	CPT4	Procedure	CPT4
2514548	99441	Non-Face-to-Face Telephone E&M Services, 5-10 mins	Procedure	Procedure	CPT4
2514549	99442	Non-Face-to-Face Telephone E&M Services, 11-20 mins	Procedure	Procedure	CPT4
2514550	99443	Non-Face-to-Face Telephone E&M Services, 21-30 mins	Procedure	Procedure	CPT4
2514551	99444	Brief check-in or e-visit	Procedure	Procedure	CPT4
36716324	722295003	Step down change in telehealth monitoring	Procedure	Procedure	SNOMED
37396332	716101005	Discussion about telehealth monitoring	Procedure	Procedure	SNOMED
40659402	G0427	Telehealth consultation emergency department or initial inpatient typically 70 minutes or more communicating with the patient via telehealth	HCPCS	Procedure	HCPCS
40663506	G0425	Telehealth consultation emergency department or initial inpatient typically 30 minutes communicating with the patient via telehealth	HCPCS	Procedure	HCPCS
40663507	G0426	Telehealth consultation emergency department or initial inpatient typically 50 minutes communicating with the patient via telehealth	HCPCS	Procedure	HCPCS
44786387	G0459	Inpatient telehealth pharmacologic management including prescription use and review of medication with no more than minimal medical psychotherapy	HCPCS	Procedure	HCPCS

© 2024 Hung P et al. *JAMA Network Open*.

eTable 3. Maternal Characteristics of Study Cohort

	Number (Column %) of Pregnancies or Median (Interquartile Range)
**All Pregnancies Given Birth from June 2018-May 2022**	349,682 (100%)
**Number of Prenatal Care Visits, Median (Interquartile)**	14 (6-22)
**Number of Prenatal Care Visits – In Person, Median (Interquartile)**	13 (5-21)
**Number of Prenatal Care Visits via Telehealth *among Telehealth Users*, Median (Interquartile)**	2 (1-4)
**Residence Location**	
Urban	228,899 (65.5%)
Rural	31,011 (8.9%)
Missing	89,772 (25.7%)
**Maternal Race and Ethnicity**	
Hispanic/Latino	59,837 (17.1%)
Non-Hispanic Groups:	
Asian	14,803 (4.2%)
Black	65,571 (18.8%)
White	162,677 (46.5%)
Other	46,794 (13.4%)
**Mother's Age, Years**	
15-19	17,936 (5.1%)
20-24	58,640 (16.8%)
25-29	93,765 (26.8%)
30-34	107,710 (30.8%)
35-39	57,978 (16.6%)
40-49	13,653 (3.9%)
**Pre-Pregnancy Body Mass Index**	
Underweight or Healthy Weight ($\leq$25.0 kg/m^2)	242,419 (69.3%)
Overweight/Obesity (> 25.0 kg/m^2)	107,263 (30.7%)
**Smoking during Pregnancy**	34,259 (9.8%)
**Preexisting or Gestational Diabetes**	41,973 (12.0%)
**Preexisting or Pregnancy-Induced Hypertension**	53,367 (15.3%)
**Depression and/or Anxiety During Pregnancy**	53,931 (15.4%)
**SARS-CoV-2 Infection during Pregnancy**	33,324 (9.5%)
**Plurality**	
Singleton	332,472 (95.1%)
Multiple	17,210 (4.9%)
**Gestational Age at Childbirth**	
Very preterm ($\leq$28 weeks)	4,634 (1.3%)
Preterm (29-36 weeks)	35,043 (10.0%)
Full term ($\geq$37 weeks)	310,005 (88.7%)
**Mode of Delivery**	
Any cesarean delivery	96,921 (27.7%)
Vaginal delivery only	252,761 (72.3%)
**Census Region**	
Northeast	48,889 (14.0%)
Midwest	93,501 (26.7%)
South	120,454 (34.4%)
West	15,829 (4.5%)

© 2024 Hung P et al. *JAMA Network Open*.

eFigure 2. Temporal Trends of Hybrid Prenatal Care and Prenatal Care Frequency among US Pregnant Individuals

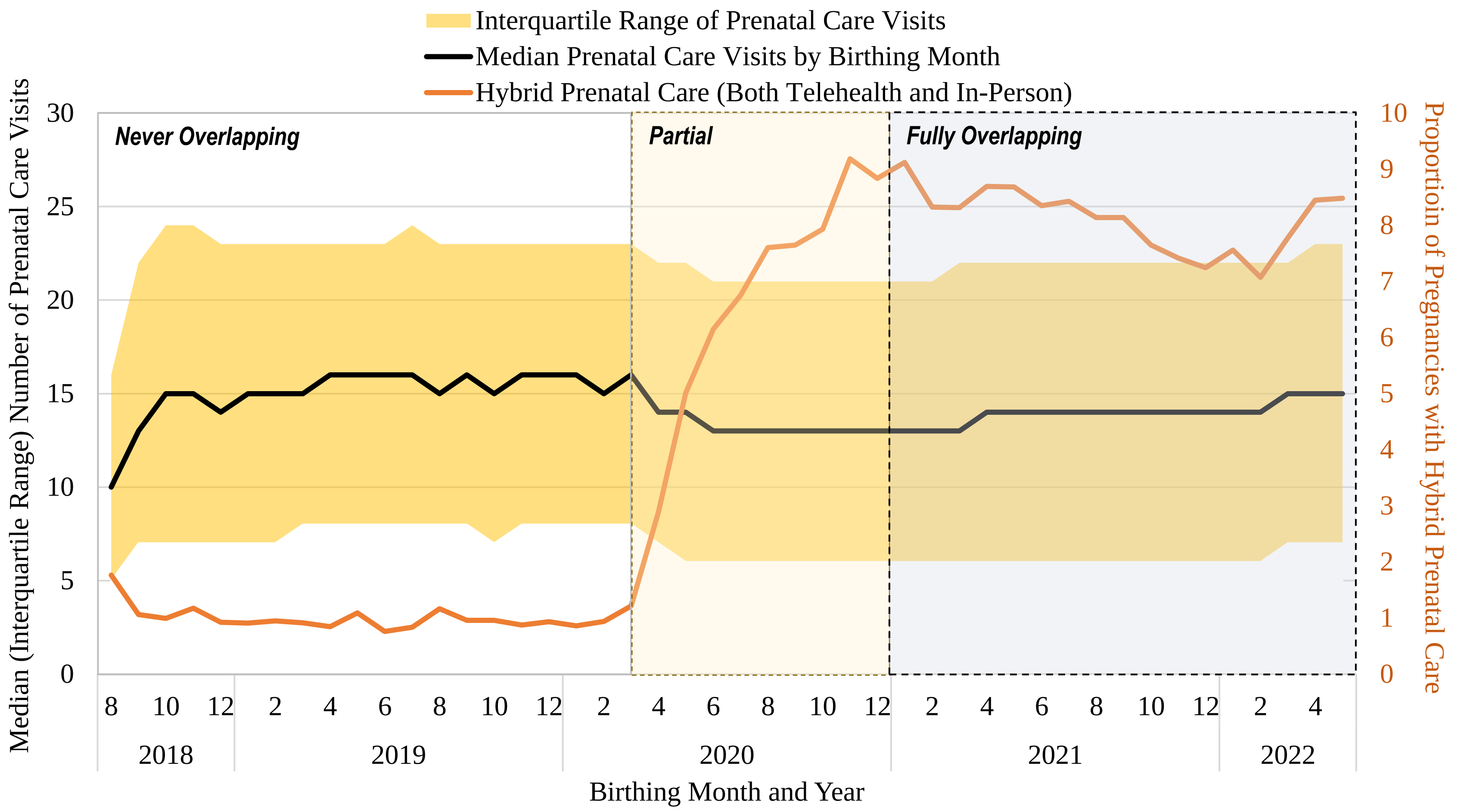

Notes: The black curve illustrates median trend in number of prenatal care visits per month of birth delivery. The orange curve shows percentages of birthing individuals with hybrid prenatal care (both telehealth and in-person visits) per month of birth delivery. There are 349,682 childbirths from all 50 states in the United States.

© 2024 Hung P et al. *JAMA Network Open*.

eFigure 3. Frequency of Pregnancies Initiating Prenatal Care and Prenatal Telehealth Visits by Gestational Week

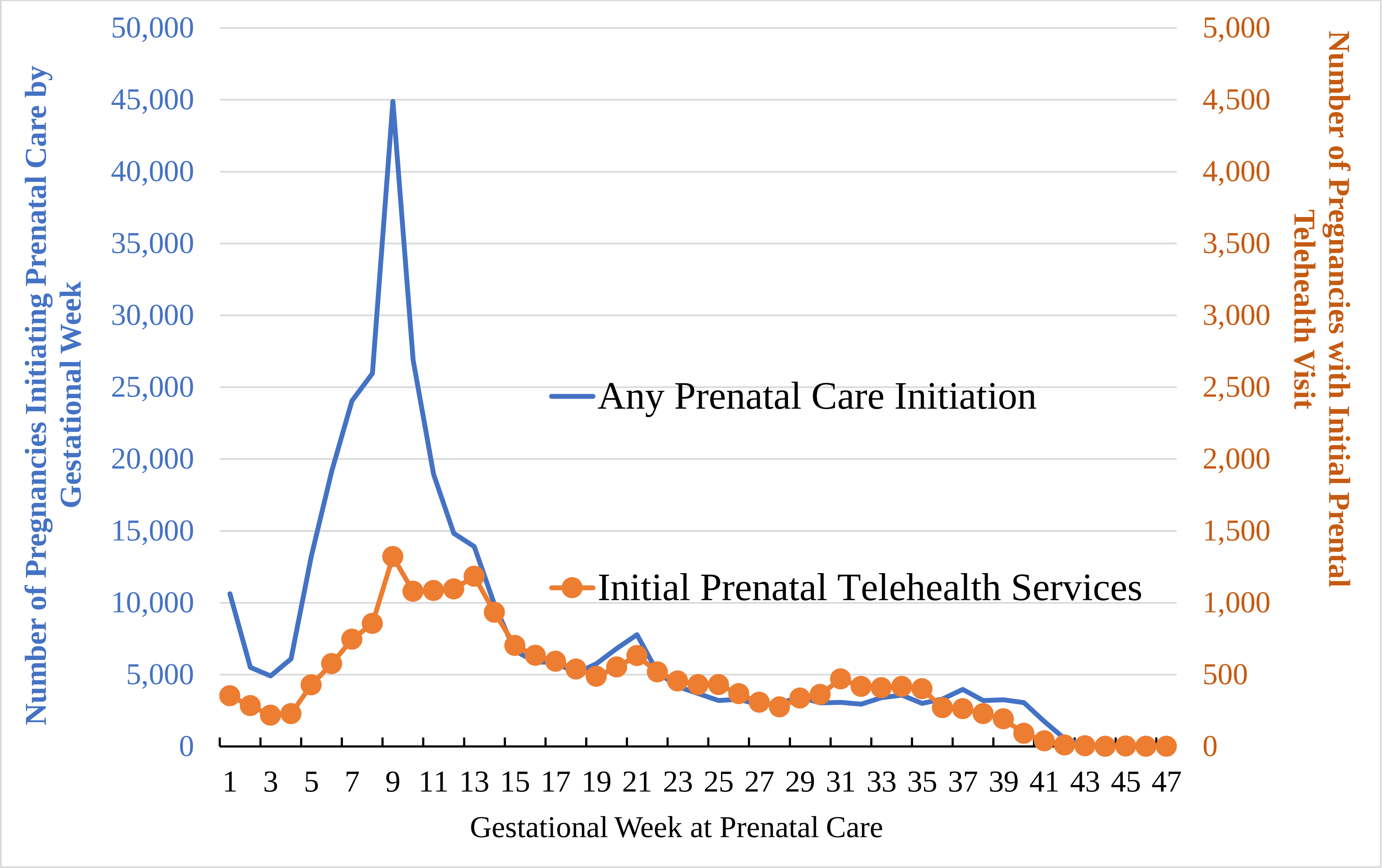

© 2024 Hung P et al. *JAMA Network Open*.

eTable 4. Distributions of Prenatal Care Utilization by Maternal Residence Rurality, Race/Ethnicity, and Age at Childbirth among Birthing People Giving Birth from June 2018 to May 2022

	Never Overlapping				Partially Overlapping				Fully Overlapping			
	Number of Prenatal Care Visits *Median (Q1-Q3)*	P value	% of Prenatal Care Visits via Telehealth *Mean (SD)*	P value	Number of Prenatal Care Visits *Median (Q1-Q3)*	P value	% of Prenatal Care Visits via Telehealth *Mean (SD)*	P value	Number of Prenatal Care Visits *Median (Q1-Q3)*	P value	% of Prenatal Care Visits via Telehealth *Mean (SD)*	P value
All Study Childbirths	15 (8-23)		0.42 (7.59)		13 (6-22)		1.88 (12.68)		13 (6-22)		2.22 (15.99)	
**Urban/Rural Residence**		<.001		<.001		<.001		<.001		<.001		<.001
Urban	16 (8-23)		0.62 (9.19)		14 (6-21)		2.41 (13.60)		14 (6-22)		2.69 (17.39)	
Rural	15 (7-22)		0.06 (3.65)		15 (6-22)		0.78 (4.98)		14 (6-22)		1.00 (7.37)	
Missing	14 (6-24)		0.00 (0.10)		12 (5-22)		1.07 (12.04)		12 (6-22		1.40 (14.30)	
**Race/Ethnicity**		<.001		<.001		<.001		<.001		<.001		<.001
Hispanic	14 (7-21)		0.51 (12.63)		11 (6-19)		3.13 (17.43)		12 (6-20)		3.53 (23.45)	
Non-Hispanic:												
Asian	16 (8-24)		0.26 (3.78)		14 (6-22)		2.37 (14.57)		14 (7-22)		2.91 (15.15)	
Black	15 (7-24)		0.78 (8.65)		14 (6-22)		2.17 (14.92)		14 (6-23)		2.38 (14.82)	
White	16 (8-24)		0.30 (5.62)		14 (6-22)		1.29 (8.86)		15 (6-23)		1.68 (13.00)	
Others[a]	16 (8-23)		0.24 (5.22)		14 (6-21)		1.70 (13.35)		13 (6-21)		1.76 (14.25)	
**Maternal Age**		<.001		0.006		<.001		<.001		<.001		<.001
15-19	14 (7-21)		0.68 (9.80)		12 (5-19)		1.49 (12.02)		12 (6-20)		1.68 (13.23)	
20-24	15 (7-22)		0.48 (6.49)		13 (6-20)		1.60 (11.41)		13 (6-21)		1.70 (14.14)	
25-29	15 (8-23)		0.39 (6.41)		14 (6-22)		1.64 (11.67)		14 (6-22)		2.02 (15.79)	
30-34	16 (8-24)		0.34 (5.52)		14 (6-22)		1.85 (11.70)		15 (6-23)		2.25 (16.09)	
35-39	15 (8-23)		0.39 (6.38)		12 (6-21)		2.41 (15.65)		13 (6-21)		2.82 (16.69)	
40-44	15 (8-26)		0.81 (24.57)		13 (6-23)		2.91 (15.49)		14 (6-22)		3.37 (19.97)	
45-49	16 (9-26.5)		0.00 (0.00)		15 (6-22)		3.89 (27.66)		15 (7-25)		5.25 (43.51)	

Notes. Q1: the 25th percentile; Q3: the 75th percentile. P values were calculated to compare median numbers of prenatal care visits by maternal race/ethnicity and age using Kruskal-Wallis tests and to compare distributions in the percentage of prenatal care visits via telehealth across pregnancies using Analysis of Variance tests. a.Others' group includes individuals identifying as American Indian or Alaska Native, multiracial, Native Hawaiian or Other Pacific Islander, or those with unknown or unreported race.

© 2024 Hung P et al. *JAMA Network Open*.

eTable 5. Differential Associations of Maternal Residence Location, Race/Ethnicity with Hybrid Prenatal Care by Prenatal Pandemic Exposure to the COVID-19 Pandemic

		Adjusted Odds Ratio (95% CI)	*P* Values[a]
**COVID-19 Pandemic Exposure During Pregnancy among Rural Non-Hispanic White Group**			
Never Overlapping		Ref	
Partial Overlapping		64.22 (31.82-129.63)	<.001
Full Overlapping		66.30 (32.97-133.34)	<.001
**Maternal Residence by Pandemic Exposure**			
**Never Overlapping**	Urban	12.23 (6.09-24.57)	<.001
	Rural	Ref	
	Missing	0.03 (0.01-0.16)	<.001
**Partial Overlapping**	Urban	1.59 (1.47-1.58)	<.001
	Rural	Ref	
	Missing	0.13 (0.14-0.20)	<.001
**Full Overlapping**	Urban	1.96 (1.97-2.01)	<.001
	Rural	Ref	
	Missing	0.19 (0.22-0.30)	<.001
**Maternal Race/Ethnicity by Pandemic Exposure**			
**Never Overlapping**	Hispanic/Latino	1.23 (0.99-1.52)	0.057
	Non-Hispanic:		
	Asian	1.03 (0.70-1.54)	0.865
	Black	1.98 (1.70-2.32)	<.001
	White	Ref	
	Others[b]	0.72 (0.53-0.98)	0.037
**Partial Overlapping**	Hispanic/Latino	1.40 (1.38-1.41)	<.001
	Non-Hispanic:		
	Asian	1.30 (1.28-1.33)	<.001
	Black	1.17 (1.16-1.19)	<.001
	White	Ref	
	Others[b]	0.86 (0.85-0.87)	<.001
**Full Overlapping**	Hispanic/Latino	1.34 (1.34-1.34)	<.001
	Non-Hispanic:		
	Asian	1.41 (1.42-1.44)	<.001
	Black	1.17 (1.18-1.19)	<.001
	White	Ref	
	Others[b]	0.84 (0.84-0.85)	<.001
**Mother's Age, Years**			
15-19		0.81 (0.75-0.87)	<.001
20-24		0.87 (0.83-0.91)	<.001
25-29		Ref	
30-34		1.15 (1.11-1.20)	<.001
35-39		1.18 (1.13-1.23)	<.001
40-49		1.24 (1.15-1.33)	<.001
**Overweight/Obesity (> 25.0 kg/m^2)**		1.64 (1.59-1.69)	<.001
**Preexisting/Gestational Diabetes**		1.49 (1.43-1.55)	<.001
**Multiple Birth vs. Singleton**		0.68 (0.63-0.74)	<.001
**Preexisting/Pregnancy-Induced Hypertension**		1.09 (1.04-1.13)	<.001
**Depression and Anxiety During Pregnancy**		1.38 (1.33-1.43)	<.001

© 2024 Hung P et al. *JAMA Network Open*.

**Smoking (Tobacco, Vaping etc.) During Pregnancy**	1.26 (1.21-1.32)	<.001
**Gestational Age at Childbirth**		
Very preterm (≤28 weeks)	Ref	
Preterm (29-36 weeks)	1.11 (0.96-1.29)	0.146
Full term (≥37 weeks)	1.37 (1.19-1.57)	<.001
**Region**		
Northeast	1.56 (1.50-1.62)	<.001
Midwest	1.13 (1.09-1.18)	<.001
South	Ref	
West	0.56 (0.52-0.61)	<.001
Unknown	3.23 (2.83-3.68)	<.001

Notes: a. p-values and adjusted odds ratios were calculated from the generalized estimating equation (GEE) with a binomial distribution and logit-link function at pregnancy level of receiving hybrid prenatal care (combined telehealth and in-person prenatal visits) versus all prenatal care visits via in-person care. b.Others' group includes individuals identifying as American Indian or Alaska Native, multiracial, Native Hawaiian or Other Pacific Islander, or those with unknown or unreported race.

© 2024 Hung P et al. *JAMA Network Open*.

eTable 6. Associations of Maternal Residence Location, Race/Ethnicity with Hybrid Prenatal Care among Pregnancies Fully Exposed to the COVID-19 Pandemic during Prenatal Period

	Adjusted Odds Ratio (95% CI)	*P* Values[a]
**Maternal Residence Location**		
Urban	1.98 (1.84-2.12)	<.001
Rural	Ref	
Missing	0.2 (0.17-0.24)	<.001
**Maternal Race/Ethnicity**		
Hispanic/Latino	1.48 (1.41-1.56)	0.057
Non-Hispanic:		
Asian	1.47 (1.35-1.59)	0.865
Black	1.18 (1.12-1.24)	<.001
White	Ref	
Other[b]	0.90 (0.84-0.96)	0.037
**Mother's Age, Years**		
15-19	0.79 (0.72-0.87)	<.001
20-24	0.85 (0.80-0.90)	<.001
25-29	Ref	
30-34	1.19 (1.13-1.24)	<.001
35-39	1.32 (1.25-1.39)	<.001
40-49	1.36 (1.25-1.49)	<.001
**Overweight/Obesity (> 25.0 kg/m^2)**	1.44 (1.38-1.49)	<.001
**Preexisting/Gestational Diabetes**	1.37 (1.3-1.43)	<.001
**Multiple Birth vs. Singleton**	0.63 (0.57-0.7)	<.001
**Preexisting/Pregnancy-Induced Hypertension**	1.04 (1.00-1.10)	0.074
**Depression and Anxiety During Pregnancy**	1.35 (1.29-1.42)	<.001
**Smoking (Tobacco, Vaping etc.) During Pregnancy**	1.32 (1.25-1.40)	<.001
**Gestational Age at Childbirth**		
Very preterm (≤28 weeks)	Ref	
Preterm (29-36 weeks)	0.99 (0.84-1.18)	0.953
Full term (≥37 weeks)	1.1 (0.94-1.29)	0.229
**Prenatal SARS-CoV-2 Infection**	1.07 (1.02-1.12)	0.003
**Region**		
Northeast	1.17 (1.12-1.24)	<.001
Midwest	0.95 (0.91-0.99)	0.017
South	Ref	
West	0.49 (0.44-0.54)	<.001
Unknown	3.01 (2.56-3.55)	<.001

Notes: a. p-values and adjusted odds ratios were calculated from the generalized estimating equation (GEE) with a binomial distribution and logit-link function at pregnancy level of receiving hybrid prenatal care (combined telehealth and in-person prenatal visits) versus exclusive in-person prenatal care. b.Others' group includes individuals identifying as American Indian or Alaska Native, multiracial, Native Hawaiian or Other Pacific Islander, or those with unknown or unreported race.

© 2024 Hung P et al. *JAMA Network Open*.
