# Supplementary material for: Racial and Ethnic and Rural Variations in the Use of Hybrid Prenatal Care in the US
Source: JAMA Netw Open. 2024 Dec 6;7(12):e2449243. doi: 10.1001/jamanetworkopen.2024.49243 (PMC11624583; doi:10.1001/jamanetworkopen.2024.49243)
Supplement: Supplement 2. — Nonauthor Collaborators. National COVID Cohort Collaborative Consortium [file jamanetwopen-e2449243-s002.pdf]

***Group Name(s): the National COVID Cohort Collaborative Consortium**

***First Name and Middle Initial(s)**	**Last Name**	***Suffix (eg, Jr, III)**	Academic Degrees	Institution	Location (city, state/province, country)	Role or Contribution, eg, chair, principal investigator	Group (if more than 1 Group listed in the byline) and/or Subgroup (eg, Steering Committee)
Melissa A.	Haendel					N3C Lead Investigators	
Christopher G.	Chute					N3C Lead Investigators	
Dave	Eichman					N3C Lead Investigators	
Anita	Walden					N3C Lead Investigators	
Emily	Pfaff					N3C Lead Investigators	
Richard	Moffit					N3C Lead Investigators	
Kenneth R.	Gersing					NCATS N3C Science Officer	
Joni L.	Rutter					NCATS N3C Leadership Team	
Kenneth R.	Gersing					NCATS N3C Leadership Team	
Penny Wung	Burgoon					NCATS N3C Leadership Team	
Samuel	Bozzette					NCATS N3C Leadership Team	
Mariam	Deacy					NCATS N3C Leadership Team	
Christopher	Dillon					NCATS N3C Leadership Team	
Rebecca	Erwin-Cohen					NCATS N3C Leadership Team	
Nicole	Garbarini					NCATS N3C Leadership Team	
Valery	Gordon					NCATS N3C Leadership Team	
Michael G.	Kurilla					NCATS N3C Leadership Team	
Emily Carlson	Marti					NCATS N3C Leadership Team	
Sam G.	Michael					NCATS N3C Leadership Team	
Lili M.	Portilla					NCATS N3C Leadership Team	
Clare	Schmitt					NCATS N3C Leadership Team	
Meredith	Temple-O'Connor					NCATS N3C Leadership Team	
Christopher G.	Chute					Data Ingest and Harmonization Team	
Emily R.	Pfaff					Data Ingest and Harmonization Team	
Davera	Gabriel					Data Ingest and Harmonization Team	
Stephanie S.	Hong					Data Ingest and Harmonization Team	
Kristin	Kostka					Data Ingest and Harmonization Team	
Harold P.	Lehmann					Data Ingest and Harmonization Team	
Richard A.	Moffitt					Data Ingest and Harmonization Team	
Michele	Morris					Data Ingest and Harmonization Team	
Matvey B.	Palchuk					Data Ingest and Harmonization Team	
Xiaohan	Tanner Zhang					Data Ingest and Harmonization Team	
Richard L.	Zhu					Data Ingest and Harmonization Team	
Emily R.	Pfaff					Phenotype Team (Individuals who create the scripts that the sites use to submit their data)	
Benjamin	Amor					Phenotype Team (Individuals who create the scripts that the sites use to submit their data)	
Mark M.	Bissell					Phenotype Team (Individuals who create the scripts that the sites use to submit their data)	
Marshall	Clark					Phenotype Team (Individuals who create the scripts that the sites use to submit their data)	
Andrew T.	Girvin					Phenotype Team (Individuals who create the scripts that the sites use to submit their data)	
Stephanie S.	Hong					Phenotype Team (Individuals who create the scripts that the sites use to submit their data)	
Kristin	Kostka					Phenotype Team (Individuals who create the scripts that the sites use to submit their data)	
Adam M.	Lee					Phenotype Team (Individuals who create the scripts that the sites use to submit their data)	
Robert T.	Miller					Phenotype Team (Individuals who create the scripts that the sites use to submit their data)	
Michele	Morris					Phenotype Team (Individuals who create the scripts that the sites use to submit their data)	
Matvey B.	Palchuk					Phenotype Team (Individuals who create the scripts that the sites use to submit their data)	
Kellie M.	Walters					Phenotype Team (Individuals who create the scripts that the sites use to submit their data)	
Johanna	Loomba					Logic Liaison Core Workgroup	
Richard A.	Moffitt					Logic Liaison Core Workgroup	
Alfred	(Jerrod) Anzalone					Logic Liaison Core Workgroup and N3C Domain Team Leads	
Evan	French					Logic Liaison Core Workgroup	
Steven G.	Johnson					Logic Liaison Core Workgroup	
Amy	Olex					Logic Liaison Core Workgroup	
Umit	Topaloglu					Logic Liaison Core Workgroup	
Andrea	Zhou					Logic Liaison Core Workgroup	
Meredith	Adams					N3C Domain Team Leads	
G.	Caleb Alexander					N3C Domain Team Leads	
Benjamin	Bates					N3C Domain Team Leads	
Will	Beasley					N3C Domain Team Leads	
Tellen D.	Bennett					N3C Domain Team Leads	
Eilis	Boudreau					N3C Domain Team Leads	
Carolyn T.	Bramante					N3C Domain Team Leads	
Donald E.	Brown					N3C Domain Team Leads	
John B.	Buse					N3C Domain Team Leads	
Tiffany J.	Callahan					N3C Domain Team Leads	
Kenrick	Cato					N3C Domain Team Leads	
Scott	Chapman					N3C Domain Team Leads	
Christopher G.	Chute					N3C Domain Team Leads	
Jaylyn	Clark					N3C Domain Team Leads	
Lesley	Cottrell					N3C Domain Team Leads	
Karen	Crowley					N3C Domain Team Leads	
Joel	Gagnier					N3C Domain Team Leads	
Jin	Ge					N3C Domain Team Leads	
Melissa A.	Haendel					N3C Domain Team Leads	
J.W.	Awori Hayanga					N3C Domain Team Leads	
Brian	Hendricks					N3C Domain Team Leads	
Elaine L.	Hill					N3C Domain Team Leads	
William	Hillegass					N3C Domain Team Leads	
Dan	Housman					N3C Domain Team Leads	
Robert	Hurley					N3C Domain Team Leads	
Jessica	Yasmine Islam					N3C Domain Team Leads	
Randeep	Jawa					N3C Domain Team Leads	
Rishikesan	Kamaleswaran					N3C Domain Team Leads	
Farrukh M.	Koraishy					N3C Domain Team Leads	
Harold P.	Lehmann					N3C Domain Team Leads	
Johanna	Loomba					N3C Domain Team Leads	
Charisse	Madlock-Brown					N3C Domain Team Leads	
Sandeep K.	Mallipattu					N3C Domain Team Leads	
Greg	Martin					N3C Domain Team Leads	
Jomol	Mathew					N3C Domain Team Leads	
Diego	Mazzotti					N3C Domain Team Leads	
Hemalkumar B.	Mehta					N3C Domain Team Leads	
Richard A.	Moffitt					N3C Domain Team Leads	
Kimberly	Murray					N3C Domain Team Leads	
Lavance	Northington					N3C Domain Team Leads	
Amy	Olex					N3C Domain Team Leads	
Shawn T.	O'Neil					N3C Domain Team Leads	
Brijesh	Patel					N3C Domain Team Leads	
Rena C.	Patel					N3C Domain Team Leads	
Emily R.	Pfaff					N3C Domain Team Leads	
Jami	Pincavitch					N3C Domain Team Leads	
Fred	Prior					N3C Domain Team Leads	
Saiju	Pyarajan					N3C Domain Team Leads	
Lee	Pyles					N3C Domain Team Leads	
Ofer	Sadan					N3C Domain Team Leads	
Nasia	Safdar					N3C Domain Team Leads	
Soko	Setoguchi					N3C Domain Team Leads	
Noha	Sharafeldin					N3C Domain Team Leads	
Anjali	Sharathkumar					N3C Domain Team Leads	
George	Sokos					N3C Domain Team Leads	
Andrew M.	Southerland					N3C Domain Team Leads	
Vignesh	Subbian					N3C Domain Team Leads	
Cliff	Takemoto					N3C Domain Team Leads	
Umit	Topaloglu					N3C Domain Team Leads	
Cavin	Ward-Caviness					N3C Domain Team Leads	
Adam B.	Wilcox					N3C Domain Team Leads	
Ken	Wilkins					N3C Domain Team Leads	
Andrew E.	Williams					N3C Domain Team Leads	
Anita	Walden					N3C Community Project Management and Operations Team	
Sruthi	Magesh					N3C Community Project Management and Operations Team	
Patricia A.	Francis					N3C Community Project Management and Operations Team	
Alexis	Graves					N3C Community Project Management and Operations Team	
Julie A.	McMurry					N3C Community Project Management and Operations Team	
Shawn T.	O'Neil					N3C Community Project Management and Operations Team	
Benjamin	Amor					Analytics Team (Individuals who build the Enclave infrastructure, help create codesets, variables, and help Domain Teams and project teams with their datasets)	
Mark M.	Bissell					Analytics Team (Individuals who build the Enclave infrastructure, help create codesets, variables, and help Domain Teams and project teams with their datasets)	

Katie Rebecca	Bradwell	Analytics Team (Individuals who build the Enclave infrastructure, help create codesets, variables, and help Domain Teams and project teams with their datasets)
Andrew T.	Girvin	Analytics Team (Individuals who build the Enclave infrastructure, help create codesets, variables, and help Domain Teams and project teams with their datasets)
Amin	Manna	Analytics Team (Individuals who build the Enclave infrastructure, help create codesets, variables, and help Domain Teams and project teams with their datasets)
Gianna	Beck	Analytics Team (Individuals who build the Enclave infrastructure, help create codesets, variables, and help Domain Teams and project teams with their datasets)
Andrew	Barros	Publication Committee
Will	Beasley	Publication Committee
Carolyn T.	Bramante	Publication Committee
Christopher G.	Chute	Publication Committee
Ran	Dai	Publication Committee
Mary	Emmett	Publication Committee
Jin	Ge	Publication Committee
Melissa A.	Haendel	Publication Committee
Jeremy	Richard Harper	Publication Committee
Wenndy	Hernandez	Publication Committee
Farrukh M.	Koraishy	Publication Committee
Meng-Hao	Li	Publication Committee
Julie A.	McMurry	Publication Committee
Hemalkumar B.	Mehta	Publication Committee
Nirup	Menon	Publication Committee
Amit	Saha	Publication Committee
Jasvinder A.	Singh	Publication Committee
Anita Wa	Walden	Publication Committee
