# Supplementary material for: Racial and Ethnic and Rural Variations in the Use of Hybrid Prenatal Care in the US
Source: JAMA Netw Open. 2024 Dec 6;7(12):e2449243. doi: 10.1001/jamanetworkopen.2024.49243 (PMC11624583; doi:10.1001/jamanetworkopen.2024.49243)
Supplement: Supplement 3. — Data Sharing Statement [file jamanetwopen-e2449243-s003.pdf]

Data Sharing Statement

Hung. Racial and Ethnic and Rural Variations in the Use of Hybrid Prenatal Care in the US. *JAMA Netw Open*. Published December 06, 2024. doi:10.1001/jamanetworkopen.2024.49243

Data
Data available: Yes
Data types: Data dictionary, Other (please specify)
Additional Information: The analyses described in this publication were conducted with data or tools accessed through the NCATS N3C Data Enclave https://covid.cd2h.org and N3C Attribution & Publication Policy v 1.2-2020-08-25b supported by NCATS Contract No. 75N95023D00001.
How to access data: Requests should be sent to the corresponding author via an email at hungp@mailbox.sc.edu.
When available: With publication

Supporting Documents
Document types: Statistical/analytic code
How to access documents: Researchers can access our codes on the N3C Enclave.
When available: With publication

Additional Information
Who can access the data: Researchers
Types of analyses: Any purpose
Mechanisms of data availability: Without investigator support
